# Kinetic Modeling and Multiobjective Optimization of Ibuprofen Synthesis Using Machine Learning

**DOI:** 10.1021/acsomega.4c11725

**Published:** 2025-08-08

**Authors:** Lang Xiang, Pengfei Qu

**Affiliations:** † School of Pharmacy, 66478 Nanjing University of Chinese Medicine, Nanjing 210023, China; ‡ School of Management Science and Engineering, 66462 Shandong Technology and Business University, Yantai 264005, China

## Abstract

This study presents a comprehensive application of integrated machine learning tools for modeling and optimizing the ibuprofen synthesis process. Initially, a database of 39,460 input combinations is created using chemical reaction theory and validated with experimental data. The CatBoost meta-model, optimized by the snow ablation optimizer, outperforms conventional algorithms in predicting reaction time, conversion rate, and production cost. Importance analyses through SHAP values identify critical input variables, notably, the concentration of the catalyst precursor (L_2_PdCl_2_), hydrogen ions (H^+^), and water (H_2_O), validating known catalytic principles and providing quantitative parameter guidance through data-driven analysis. Multiobjective optimization using NSGA-II generates a Pareto front of solutions, from which four industrial strategies are derived: balanced performance, maximum output, maximum yield, and minimum cost, each suitable for different production scenarios. The results identify optimal catalyst concentration ranges (0.002–0.01 mol/m^3^) that achieve high conversion rates while maintaining low costs. Uncertainty analysis conducted through Monte Carlo simulation reveals that reaction time exhibits particularly high sensitivity to parameter fluctuations, with a distinctive nonlinear response peaking at moderate perturbation levels (σ = 0.3). This study provides valuable insights for the rational design of ibuprofen synthesis conditions and demonstrates the effectiveness of integrating physics-based modeling with machine learning for chemical process optimization.

## Graphical abstract

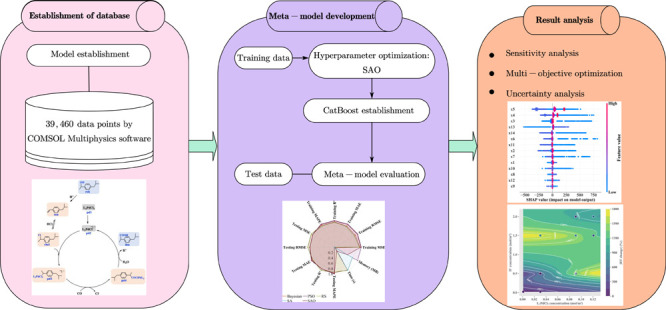

## 1 Introduction

Ibuprofen is a widely used nonsteroidal anti-inflammatory drug (NSAID) for relieving pain, reducing inflammation, and fever.^1,2^ Due to its significant pharmacological effects and market demand, the efficient synthesis of ibuprofen holds substantial research value in chemical engineering and the pharmaceutical industry.^3^ Enhancing the synthesis efficiency of ibuprofen not only meets market demands but also reduces production costs and environmental pollution, thereby having significant practical application value.^4^ Additionally, the synthesis process of ibuprofen is complex, involving multiple catalytic reaction steps that need to be conducted under specific conditions to ensure high efficiency and selectivity of the products.^5,6^ Therefore, optimizing the synthesis process of ibuprofen is of critical importance.

Although substantial research has been devoted to optimizing the synthesis of ibuprofen, several limitations still exist. First, traditional chemical synthesis methods have achieved certain successes in optimizing reaction conditions and increasing yield, but they still suffer from low efficiency, high costs, and environmental pollution.^7^ For example, traditional multistep reaction processes are complex and easily affected by changes in reaction conditions, leading to instability in product purity and yield.^8^ Second, current research, both domestically and internationally, mainly focuses on single optimization objectives, such as increasing yield or reducing reaction time, often neglecting the need for multiobjective optimization.^9^ The synthesis process of ibuprofen involves multiple critical reaction steps and complex interactions of various reaction conditions, making it difficult for single-objective optimization methods to comprehensively enhance overall synthesis efficiency.^3^ Moreover, most existing research is based on laboratory scale and lacks optimization studies under industrial production conditions.^10^ There is a significant difference between laboratory conditions and industrial production conditions, making it challenging to directly apply laboratory research results to industrial production. This is particularly important in large-scale production, where the uncertainty of reaction conditions and fluctuations in external environments can significantly impact the quality and yield of the final product.^11^ Finally, purely data-driven optimization approaches typically require extensive experimental data sets, incurring significant costs.^12^ Conversely, for reaction systems, integrating first-principle models with Design of Experiments (DoE) methodologies and metaheuristic techniques demonstrably reduces data requirements.^13,14^ Our proposed framework synergizes physics-based modeling with machine learning capabilities to overcome the limitations of either approach independently.

In recent years, the application of machine learning technology in various fields has gradually increased, demonstrating its significant advantages in handling high-dimensional and nonlinear problems.^15^ Machine learning methods have achieved major advancements in image recognition, natural language processing, financial forecasting, and other areas.^16^ Common optimization algorithms include Genetic Algorithm (GA), Particle Swarm Optimization (PSO), Simulated Annealing (SA), and Random Search (RS). These algorithms have shown strong capabilities in solving complex optimization problems, but also have some limitations. For example, GA and PSO have demonstrated success in various multimodal optimization problems when properly implemented and tuned, although they may face challenges in high-dimensional spaces without appropriate parameter adjustment. RS is generally considered less efficient in high-dimensional parameter spaces compared to guided search methods, while SA can effectively handle complex landscapes but may require careful cooling schedule tuning.^17^ Additionally, commonly used machine learning algorithms, such as support vector machine (SVM), neural networks (NN), and gradient boosting trees (GBT), have achieved significant results in various applications. However, SVM has high computational costs when handling large data sets, NN is highly dependent on large amounts of labeled data, and GBT has certain limitations in dealing with categorical features and missing values.^18^ CatBoost, as a gradient boosting machine learning algorithm, has gained widespread application in various fields in recent years due to its superiority in handling categorical features and efficient computational performance.^19^ CatBoost can effectively handle categorical features and missing values, significantly reducing training time while maintaining high accuracy.^20^ The snow ablation optimizer (SAO), similar to other metaheuristic approaches such as GA, PSO, and SA, simulates the snow melting process in physical systems, offering a balance between exploration and exploitation that aims to effectively navigate complex solution spaces while reducing the likelihood of premature convergence to local optima.^21^ While deterministic global optimization methods like Branch and Bound can guarantee optimality for certain problems, metaheuristic approaches often provide a favorable balance between computational efficiency and solution quality for applications like ibuprofen synthesis.^22^ Therefore, this study is particularly interested in applying SAO in combination with CatBoost to the optimization process of ibuprofen synthesis.

Interpretable machine learning methods include commonly used techniques, such as LIME, SHAP, and interpretable decision trees. Among them, SHAP is based on game theory and can provide both global and local explanations, offering consistency and accuracy.^23–25^ Multiobjective optimization algorithms such as NSGA-II, MOEA/D, and SPEA2 are classic methods. NSGA-II, through fast nondominated sorting and crowding distance assignment, maintains solution diversity while improving optimization efficiency, making it suitable for complex multiobjective optimization problems in ibuprofen synthesis.^26–28^ In terms of uncertainty analysis, common methods include Monte Carlo simulation, adaptive sampling, response surface methodology, and Gaussian processes. The advantage of Monte Carlo simulation lies in its ability to provide accurate probability distributions and assess the impact of input variable fluctuations on model output.^29,30^ By utilizing these auxiliary methods, this study brings new perspectives and tools to the modeling and optimization of ibuprofen synthesis, providing strong support for improving the synthesis efficiency and stability.

Recent developments in pharmaceutical process optimization have demonstrated an increasing integration of machine learning with traditional chemical engineering approaches. In this context, Quality by Design (QbD) has emerged as a systematic framework for pharmaceutical development, with comprehensive reviews analyzing its current implementation status and identifying adoption challenges across the pharmaceutical industry.^31^ Building upon QbD principles, multiobjective optimization techniques such as NSGA-II have been successfully applied to pharmaceutical manufacturing, including reliability optimization of pharmaceutical plants.^32^ Furthermore, model-based optimization and control strategies rooted in process modeling have demonstrated significant potential in advancing pharmaceutical manufacturing by reducing development times, improving productivity and quality control, and enhancing fundamental process understanding.^33^ Complementing these model-based approaches, predictive modeling and artificial intelligence-driven automation have been increasingly integrated into pharmaceutical manufacturing to enable real-time process optimization, predictive maintenance, and enhanced quality control.^34^ Similarly, data-driven modeling methods and techniques have been adopted to enable automated reaction modeling, process optimization, and predictive analysis across multiple manufacturing scales.^35^ However, despite these advances, comprehensive frameworks that simultaneously address multistep synthesis kinetics, economic optimization, and process robustness analysis remain limited in the literature, highlighting the need for integrated approaches like the one presented in this ibuprofen synthesis study.

This study aims to optimize the ibuprofen synthesis process by integrating the SAO with the CatBoost algorithm and conducting a comprehensive uncertainty analysis. The paper is structured as follows: First, the methodology section elaborates on the kinetic modeling of ibuprofen synthesis, database establishment, meta-model development algorithms, and multiobjective optimization techniques; Second, the results and discussion section presents meta-model development, sensitivity analysis, multiobjective optimization results, and uncertainty analysis; Finally, the conclusion summarizes the research findings and proposes directions for future work. While this work demonstrates the methodological framework and provides optimization insights, the results are based on simulation data and require experimental validation for practical industrial implementation.

## 2 Methodology

Based on the reaction kinetics model established in COMSOL Multiphysics software, 39,460 data points are generated. This study then applies CatBoost for predictive modeling with SAO for hyperparameter optimization, followed by NSGA-II for multiobjective optimization. Finally, the analysis of results is conducted, including sensitivity analysis, multiobjective optimization, and uncertainty analysis. Detailed information about the methodology mentioned above can be found in the Supporting Information (SI). It should be noted that this study employs COMSOL simulation data for model training and validation, which represent a computational modeling approach. The optimization strategies derived from this framework require experimental verification before industrial application.

### 2.1 Database Establishment and Data Preparation

In this study, a simulation model, implemented in COMSOL Multiphysics software, is employed to generate a data set with 39,460 data points, aimed at exploring the catalytic reaction kinetics of ibuprofen synthesis. The kinetic modeling of ibuprofen synthesis (Text S1) involves a comprehensive reaction mechanism consisting of seven key steps: alcohol dehydration, alkene hydrohalogenation, substrate dehydrohalogenation, catalyst activation, oxidative addition, carbonylation, and finally hydrolysis to form ibuprofen. Each reaction step is mathematically described with specific rate expressions, providing a solid foundation for simulating the overall process dynamics.

Table 1 summarizes the input variables used in data collection. To establish a comprehensive data set for machine learning model development, the ranges of these input variables are based on the parameter settings from the official COMSOL case library. The minimum values are set to 0.1× the reference values, and the maximum values are set to 10x the reference values. This wide parameter coverage is designed to (1) provide sufficient data diversity essential for robust machine learning model training, (2) ensure comprehensive coverage of the parameter space to capture complex nonlinear system behaviors, (3) prevent model generalization issues that arise from overly constrained parameter ranges, and (4) establish an adequate search space for effective multiobjective optimization. This approach follows established practices in machine learning for chemical engineering applications, where broad parameter exploration is necessary for developing predictive models with good generalization capabilities.^36,37^ The model simulates a multistep catalytic process using a homogeneous catalyst in a perfectly stirred batch reactor. It meticulously tracks each reaction step from the dehydration of 1-(4-isobutylphenyl)­ethanol to the formation of ibuprofen, while rate expressions and material balances are automatically generated by the Chemical Reaction Engineering module. The specific details of the modeling can be referenced from the COMSOL case study website: https://cn.comsol.com/model/ibuprofen-synthesis-1307.

**Table 1:** Summary of Input Variables for Data Collection

input variable	description	minimum value	maximum value	unit
*x*_1_	*c*_0_ (B)	0.01	1.0	mol/m^3^
*x*_2_	*c*_0_ (Cl^–^)	0.02	2.0	mol/m^3^
*x*_3_	*c*_0_ (H^+^)	0.02	2.0	mol/m^3^
*x*_4_	*c*_0_ (H_2_O)	0.3	30	mol/m^3^
*x*_5_	*c*_0_ (L_2_PdCl_2_)	0.00121	0.121	mol/m^3^
*x*_6_	*k*_1_	7.45 × 10^–4^	7.45 × 10^–2^	m^3^/(s mol)
*x*_7_	*k*_2_	1.25 × 10^–3^	1.25 × 10^–1^	m^6^/(s mol^2^)
*x*_8_	*k*_3_	1.60 × 10^–4^	1.60 × 10^–2^	m^3^/(s mol)
*x*_9_	*k*_4_	1.5 × 10^–2^	1.5	m^6^/(s mol^2^)
*x*_10_	*k*_5_	0.159	15.9	m^3^/(s mol)
*x*_11_	*k*_6_	2.14 × 10^–2^	2.14	m^3^/(s mol)
*x*_12_	*k*_7_	9.52 × 10^–2^	9.52	m^3^/(s mol)
*x*_13_	*k*_8_^f^	5 × 10^–2^	5	m^6^/(s mol^2^)
*x*_14_	*k*_8_^r^	1 × 10^–3^	1 × 10^–1^	m^6^/(s mol^2^)

As shown in Figure 1, the simulation model demonstrates excellent agreement with experimental data^38^ for the concentration–time profiles of key components in the ibuprofen synthesis process. The model accurately captures the consumption of roh, the formation and subsequent consumption of ren, and the gradual production of ibu. This validation confirms the reliability of the COMSOL model in predicting the reaction kinetics and supports the credibility of the simulation-generated data set used for subsequent optimization studies.

**Figure 1:**
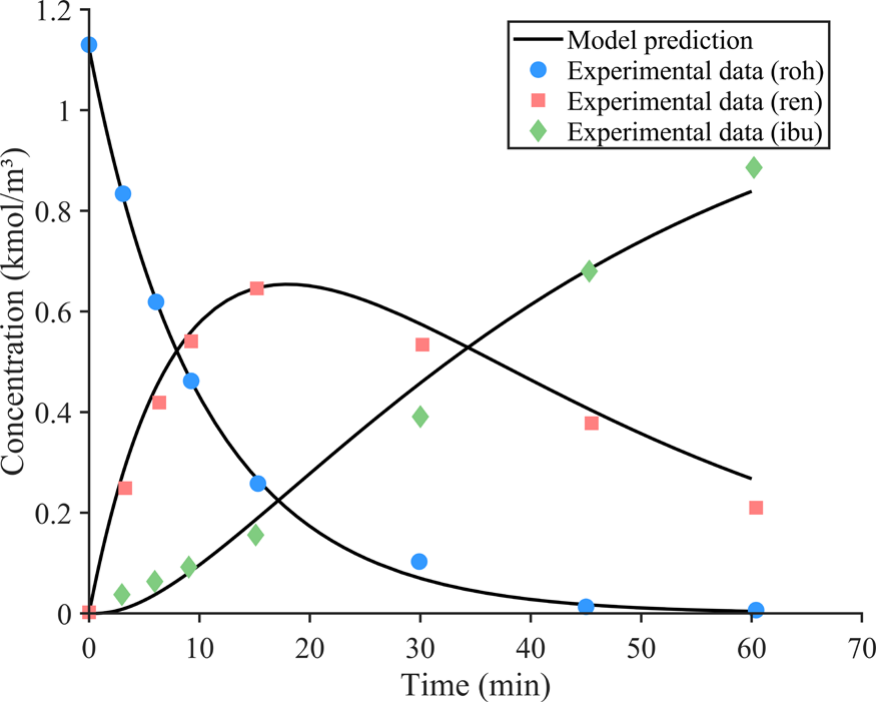
Comparison of experimental and predicted concentration–time profiles using the COMSOL dynamic model.

Moreover, the model incorporates a reversible reaction that simulates the esterification of ibuprofen with the reactant alcohol. This feature enables the observation of dynamic changes in the reaction process and product formation under various conditions, providing insights into the catalytic mechanism and its effect on product formation. The model outputs key metrics, such as reaction time (RT), conversion rate (CR), and production cost (Cost), which are essential for assessing reaction efficiency and optimizing operational conditions. In this study, RT specifically refers to the time point at which the reaction reaches a stable state (where ibuprofen production stabilizes), while CR refers to the ratio of the ibuprofen amount to the initial reactant amount at the stable state. The Cost metric represents the comprehensive economic cost of the synthesis process, calculated by considering multiple factors, including catalyst expense, raw materials, energy consumption, and purification requirements. These three metrics capture different aspects of the reaction performance: RT measures process speed, and CR measures reaction efficiency and completion degree, while Cost evaluates the economic viability of the process.

The Cost metric is calculated using a comprehensive model that accounts for various economic factors in the production process. It should be noted that this cost model is a simplified economic evaluation model designed for multiobjective optimization analysis, primarily used for comparing the relative economic performance of different process parameter combinations rather than absolute cost prediction. The parameter settings consider typical cost structures in pharmaceutical engineering, and while specific values may vary by region and scale, the relative relationships have general applicability. Calibration and verification are recommended based on specific production conditions in actual industrial applications. The model structure is constructed based on standard frameworks in chemical engineering design economics:^39,40^

$$\mathrm{Cost}=C_{\mathrm{catalyst}}+C_{\mathrm{reagent}}+C_{\mathrm{fixed}}+C_{\mathrm{energy}}+C_{\mathrm{maint}}+C_{\mathrm{purif}}+C_{\mathrm{labor}}$$ 1

where *C*_catalyst_ = 8000 × *x*_5_^1.2^ represents the catalyst cost with the base value referencing palladium-based catalyst market price levels and the exponent reflecting the nonlinear cost increase with catalyst concentration due to precious metal content, consistent with the economic principles of catalyst preparation and recovery; *C*_reagent_ = 40*x*_1_ + 20*x*_2_ + 30*x*_3_ + 10*x*_4_ accounts for the costs of various reagents, with coefficients based on typical pricing data from chemical reagent suppliers; *C*_fixed_ = 200 is the fixed operational cost, representing equipment depreciation and utility infrastructure expenses; $C_{\mathrm{energy}}=15\cdot \sqrt{y_{1}}$ captures energy consumption that increases with reaction time, with the square root relationship reflecting the marginal decreasing effect of energy consumption growth when reaction time extends; *C*_maint_ = 10 × (1 + 0.05 × *y*_1_^1.5^) represents maintenance costs associated with equipment usage duration, with exponent 1.5 simulating the accelerated accumulation process of equipment wear; *C*_purif_ = 500 × (1 – *y*_2_)^1.5^ addresses purification costs that increase significantly at lower conversion rates, with the nonlinear relationship based on chromatographic separation economics principles; and *C*_labor_ = 150 + 5 × *y*_1_ accounts for labor costs proportional to process duration.

Figure 2 presents the correlation matrix of input variables (*x*_1_–*x*_14_) and output variables (RT as *y*_1_, CR as *y*_2_, and Cost as *y*_3_). Several significant patterns emerge: (1) Input variables (*x*_1_–*x*_14_) exhibit strong positive correlations among themselves (0.65–0.69), suggesting potential multicollinearity in the data set. (2) The catalyst precursor concentration (*x*_5_) shows the strongest negative correlation with conversion rate (*y*_2_) at −0.94, confirming its critical role in the reaction efficiency. (3) Reaction time (*y*_1_) and conversion rate (*y*_2_) demonstrate a moderate positive correlation (0.41), while reaction time and cost (*y*_3_) show a stronger positive correlation (0.56), and conversion rate negatively correlates with cost (−0.44). These relationships validate treating RT, CR, and Cost as separate optimization objectives.

**Figure 2:**
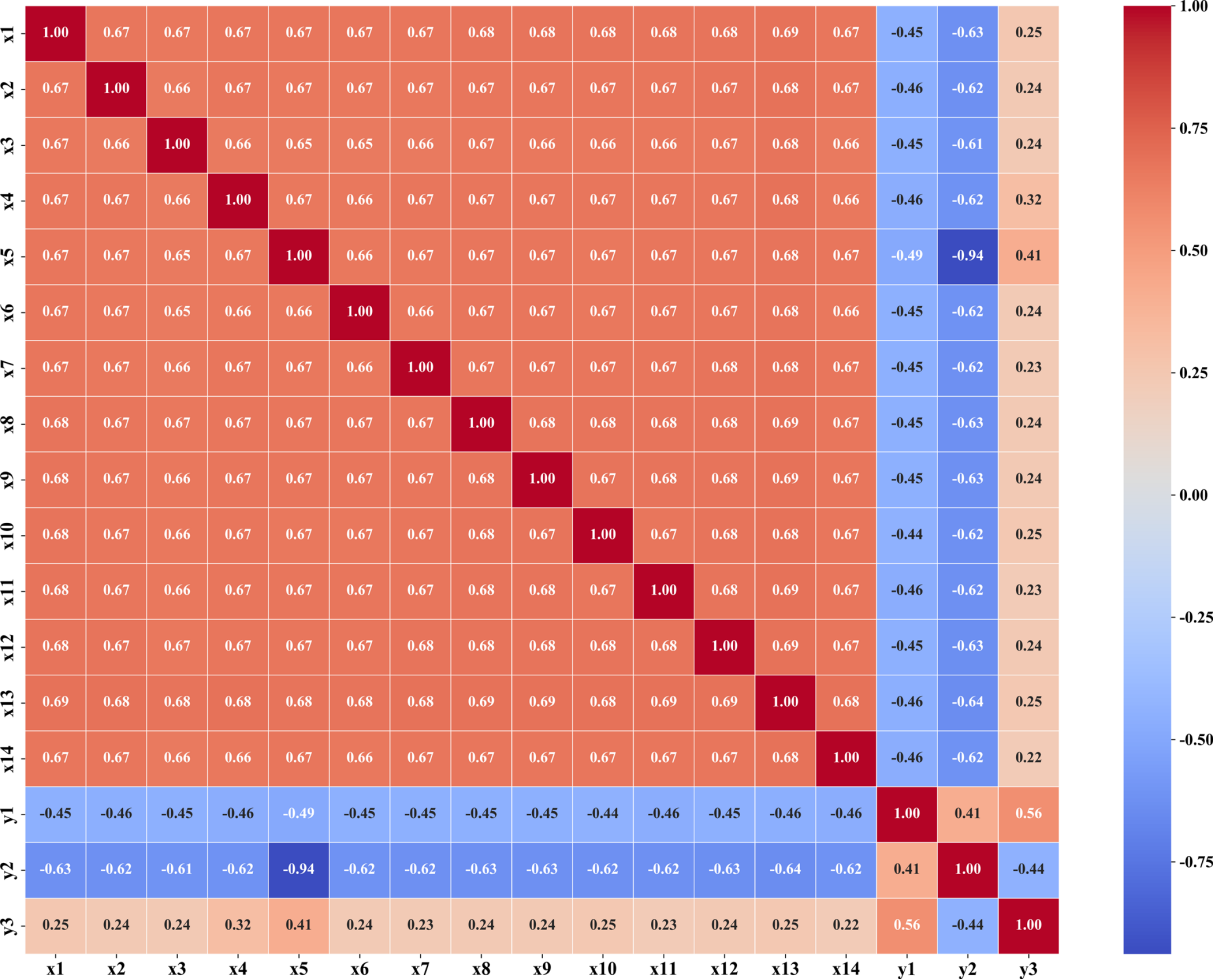
Correlation matrix of input variables (*x*_1_–*x*_14_) and output variables (RT, CR, and Cost).

### 2.2 Meta-Model Development Algorithms

For hyperparameter optimization, the SAO (Text S2) is employed, an innovative metaheuristic algorithm inspired by natural snow melting processes. SAO balances exploration and exploitation through dual mechanisms simulating snow sublimation and melting, preventing premature convergence to local optimal values while efficiently navigating complex parameter spaces.

The CatBoost algorithm (Text S3) is selected as the base prediction model due to its exceptional performance in handling heterogeneous feature sets and complex dependencies. Its innovative approach to categorical feature processing and gradient bias reduction makes it particularly suitable for modeling complex chemical synthesis processes. Model performance is evaluated using multiple statistical metrics (Text S4), including mean squared error (MSE), root mean squared error (RMSE), mean absolute error (MAE), coefficient of determination (*R* ^2^), and mean absolute percentage error (MAPE), providing a comprehensive assessment of prediction quality.

### 2.3 Multiobjective Optimization with NSGA-II

For multiobjective optimization, the NSGA-II algorithm (Text S5) is implemented, which efficiently identifies Pareto-optimal solutions through fast nondominated sorting and crowding distance calculation. This approach allows the simultaneous optimization of reaction time, conversion rate, and production cost while preserving solution diversity.

## 3 Results and Discussion

### 3.1 Meta-Model Development

#### 3.1.1 Selection of Optimization Algorithms

To ensure a fair comparison, this study implements and evaluates five hyperparameter optimization algorithms under a unified experimental framework: Random Search (RS), Bayesian Optimization (Bayesian), Particle Swarm Optimization (PSO), simulated annealing (SA), and snow ablation optimizer (SAO). All algorithms operate within identical hyperparameter search spaces (learning_rate: 0.01–0.3, depth: 4–10, l2_leaf_reg: 0–10, iterations: 100–1000), with each algorithm executed through five independent experiments, a maximum of 20 iterations, and a uniform time constraint of 1200 s. Regarding algorithmic configurations, Random Search utilizes pure random sampling; Bayesian Optimization employs the gp_minimize function to construct Gaussian process surrogate models; PSO configures a swarm size of 15 particles; Simulated Annealing implements an initial temperature of 100.0 with a cooling rate of 0.95; and SAO establishes an initial population size of 15, featuring dynamic population grouping and adaptive search intensity parameters (DDF). To guarantee evaluation fairness, all algorithms employ identical CatBoost configurations and training-validation-testing frameworks, uniformly using the mean squared error on the validation set as the optimization objective. As shown in Table 2, different algorithms exhibit significant performance variations across RT, CR, and Cost prediction tasks, with ANOVA analysis confirming the statistical significance of these differences (RT: *F* = 2.98, *p* = 0.044; Cost: *F* = 3.28, *p* = 0.032).

**Table 2:** Comparison of Optimization Algorithms on RT, CR, and Cost with Mean Performance Metrics

	testing set performance (mean values)				computational resources	
algorithm	RMSE_mean	MAE_mean	*R* ^2^_mean	MAPE_mean (%)	time_mean (s)	memory_mean (MB)
RT results						
SAO	2.085	0.306	0.995	2.462	730.61	9.49
RS	2.028	0.318	0.995	3.079	38.49	23.31
Bayesian	2.031	0.314	0.995	2.788	49.54	22.99
PSO	2.081	0.302	0.995	2.335	872.01	15.27
SA	2.722	0.581	0.991	12.354	34.82	17.11
CR results						
SAO	3.04 × 10^–4^	1.30 × 10^–5^	0.99995	0.0022	680.47	24.27
RS	3.26 × 10^–4^	1.90 × 10^–5^	0.99994	0.0030	36.74	18.98
Bayesian	3.36 × 10^–4^	1.80 × 10^–5^	0.99993	0.0030	41.90	22.25
PSO	3.16 × 10^–4^	1.90 × 10^–5^	0.99994	0.0028	279.18	13.60
SA	4.70 × 10^–4^	4.70 × 10^–5^	0.99984	0.0060	29.67	32.86
Cost results						
SAO	26.91	3.81	0.9940	0.314	653.14	17.86
RS	27.20	3.85	0.9938	0.324	38.86	37.26
Bayesian	27.06	3.88	0.9939	0.326	49.54	28.42
PSO	26.72	3.56	0.9941	0.292	847.99	18.66
SA	33.53	6.86	0.9903	0.635	35.75	24.85

The SAO algorithm demonstrates distinct advantages across multiple performance metrics. As illustrated in Figure 3, SAO rapidly decreases the objective function value during early iterations while maintaining a superior convergence performance. For RT prediction, although SAO’s RMSE_mean (2.085) is slightly higher than RS (2.028), its MAPE_mean (2.462%) substantially outperforms SA (12.354%); in CR prediction, SAO achieves near-perfect prediction accuracy with MAPE_mean of merely 0.0022% and *R* ^2^_mean reaching 0.99995. SAO’s performance superiority derives from its distinctive exploration-exploitation balance mechanism: the exploration phase employs stochastic perturbations based on both the global best solution and population centroid to facilitate a wide-ranging search, while the exploitation phase utilizes temperature-modulated attraction toward the best solution combined with controlled random movements to conduct precise refinement. Simultaneously, the algorithm dynamically adjusts population proportions to enhance adaptability across different phases, effectively avoiding local optima traps. Figure 4 radar charts further confirm SAO’s comprehensive advantages across multidimensional performance metrics, particularly approaching the periphery of the chart for prediction accuracy-related indicators.

**Figure 3:**
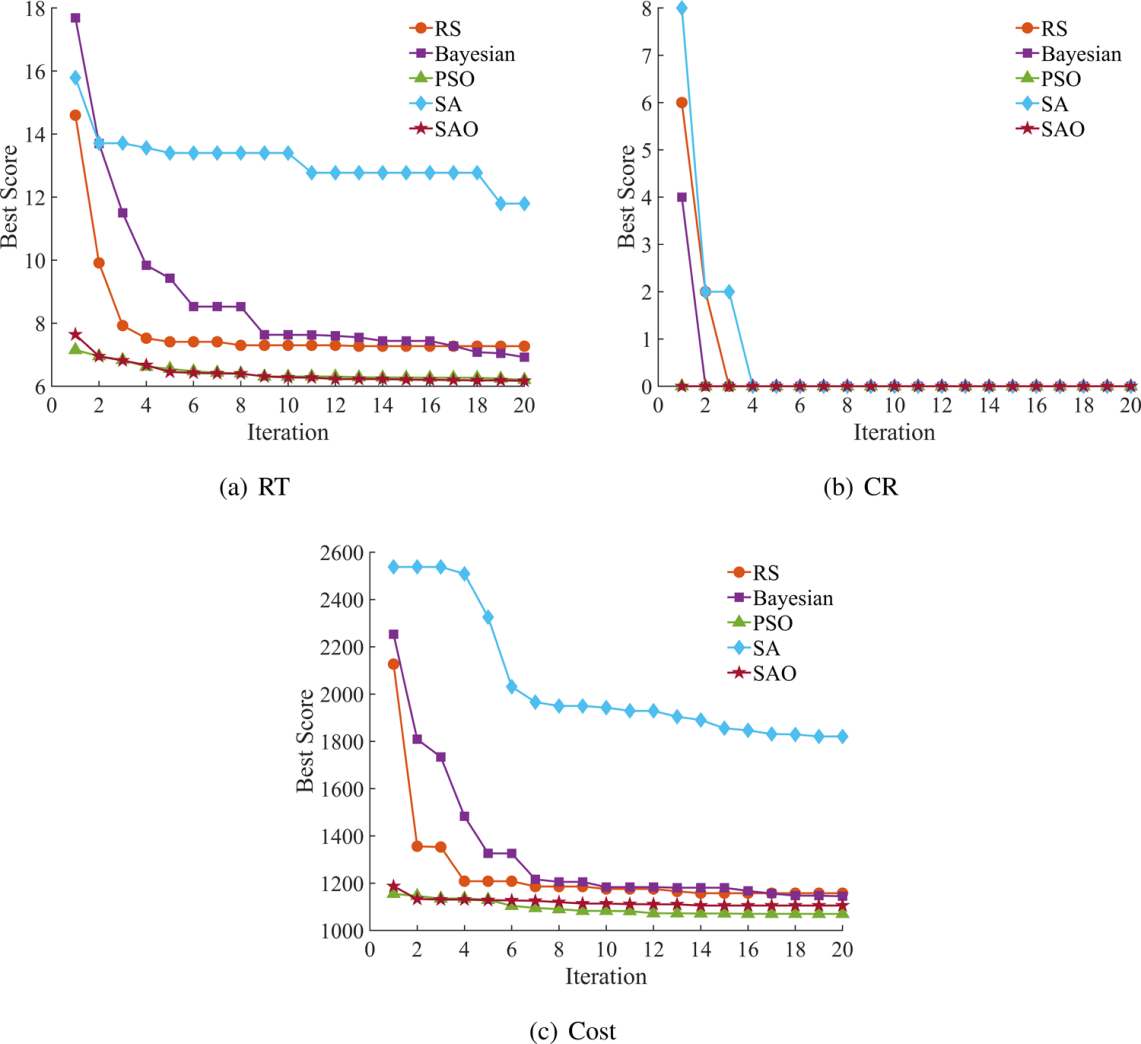
Convergence comparison of five optimization algorithms with predictions of RT, CR, and Cost.

**Figure 4:**
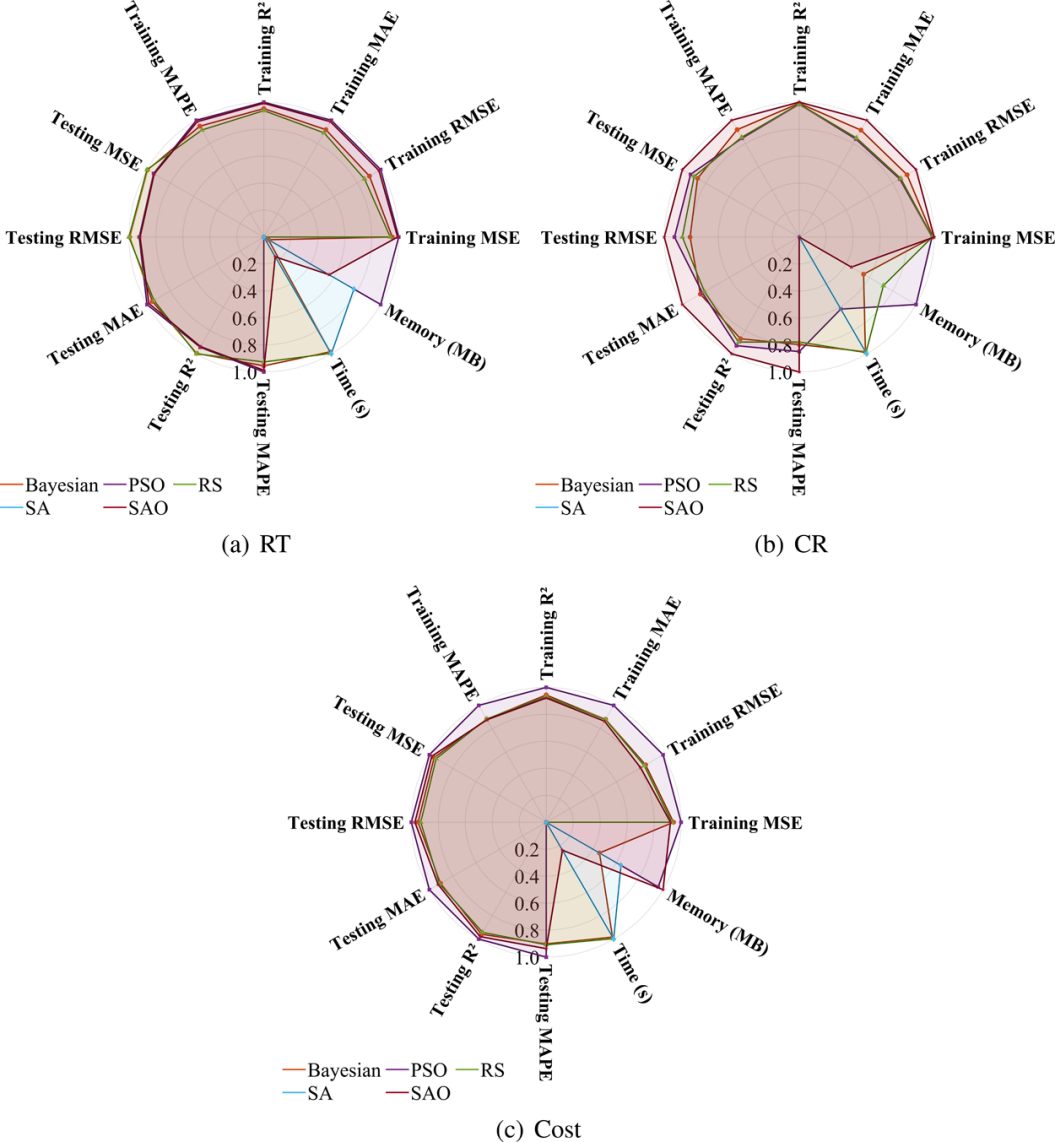
Multidimensional performance analysis of optimization algorithms.

From a computational resource perspective, as indicated in the final two columns of Table 2, SAO averages approximately 700 s across the three prediction tasks, significantly higher than SA (approximately 35 s) and RS (approximately 39 s); however, it demonstrates excellent memory utilization efficiency, requiring merely 9.49MB for RT prediction, lower than RS (23.31MB) and Bayesian (22.99MB). Despite higher computational costs, SAO delivers substantial prediction accuracy improvements: compared to SA, MAPE decreases by 80% for RT prediction, 63% for CR prediction, and 51% for Cost prediction. This “time-for-accuracy” trade-off presents practical value in ibuprofen synthesis optimization—considering the stringent requirements for prediction accuracy and stability in pharmaceutical synthesis processes, SAO’s additional computational expenditure is entirely justified, particularly in high-value industrial applications where minimal precision improvements can translate into significant economic benefits. Comprehensive evaluation indicates that SAO represents the optimal selection for CatBoost hyperparameter optimization, especially applicable in scenarios requiring high-precision predictions with sufficient computational resources.

#### 3.1.2 Assessment of Meta-Model Performance

This study uses the open-source machine learning library CatBoost, which is based on Python and can be accessed on GitHub (https://github.com/catboost/catboost). Because of its ability to handle categorical features, CatBoost demonstrates outstanding performance in both classification and regression tasks, effectively simplifying data preprocessing. The advanced features offered by this library are fully utilized to complete meta-model training and evaluation successfully. To further improve the overall performance of the meta-model, the SAO algorithm is employed to finely adjust several key hyperparameters, including depth (max_depth), learning rate, L2 leaf regularization (L2_leaf_reg), and the number of iterations. The refined hyperparameters, presented in detail in Table 3, outline the optimal configurations for various tasks. These adjustments aim to follow academic standards and improve the readability, thereby enhancing prediction accuracy.

**Table 3:** Specification of Hyperparameters for Enhanced Performance in the CatBoost Models

		optimal parameters		
parameters	value ranges	RT	CR	cost
depth (max_depth)	[4, 10]	7	7	8
learning rate	[0.01, 0.3]	0.140	0.300	0.139
L2_leaf_reg	[0, 10]	4.733	3.194	5.000
iterations	[100, 1000]	994	572	520

As shown in Figure 5, the learning curves of the meta-models employing CatBoost algorithms are presented. As training samples increase, the training and cross-validation scores for RT and CR converge and stabilize, demonstrating the models’ good generalization capabilities. The cross-validation scores for CR significantly improve with additional samples, reflecting enhanced adaptability to unseen data. The Cost index performance is particularly notable, with both training and cross-validation scores maintaining extremely high levels (near 1.0) and almost completely overlapping throughout, indicating exceptional precision in capturing economic cost relationships. The minimal gap between the training and cross-validation scores confirms the absence of overfitting across all three indices. All models maintain high scores, indicating their accuracy in predicting RT, CR, and Cost. These learning curves also reveal that as training data increases, improvements in model performance tend to saturate, suggesting the importance of optimizing computational resources and data collection strategies.

**Figure 5:**
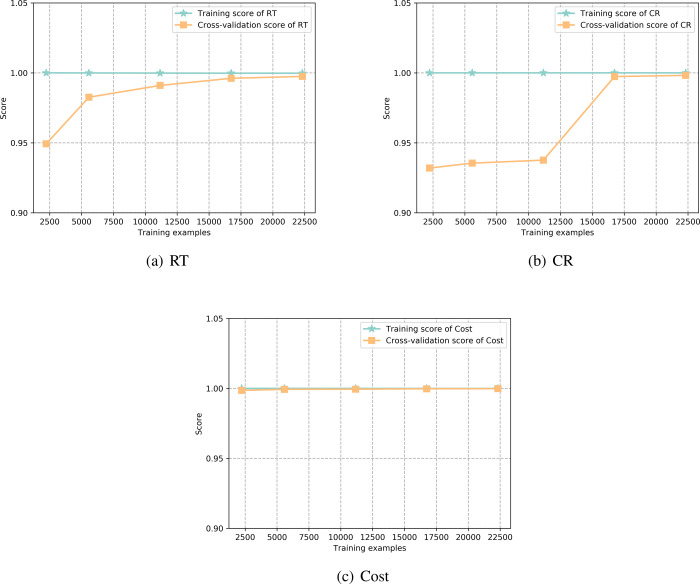
Learning curves of the meta-models utilizing CatBoost algorithms.

As shown in Figure 6, the comparison between meta-model predictions and COMSOL-based results is illustrated with best-fit lines and 95% prediction intervals. Figure 6 shows the relationships for RT, CR, and Cost, respectively. Evidently, most training and testing data points are closely distributed around the best-fit lines, indicating high consistency between the predictions and actual observations. The color gradient from blue to yellow visually represents a data density distribution, with high-density areas signifying close matches between predictions and COMSOL values.

**Figure 6:**
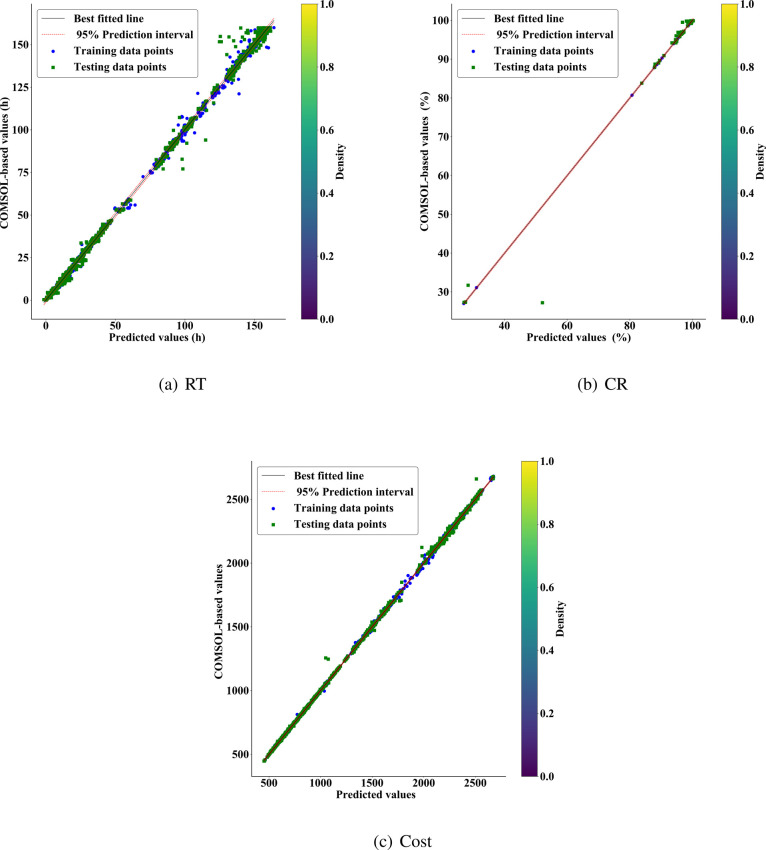
Comparisons between meta-model predictions and COMSOL-based results with the best fitted line and 95% prediction interval.

The best-fitted line expressions are represented as follows:

$$\begin{array}{l} y=1.0012\hat{y}-0.0079(\mathrm{RT})\\ y=1.0013\hat{y}-0.1239(\mathrm{CR})\\ y=1.0002\hat{y}-0.1582(\mathrm{Cost}) \end{array}$$ 2

where *y* represents COMSOL-based values and *ŷ* represents meta-model predictions. All three indices show slopes approaching unity and intercepts near zero, indicating exceptionally accurate predictions. Statistical analysis shows *t*-values of 9356.965, 6297.812, and 22400 for RT, CR, and Cost fitting equations, respectively, with *p*-values all below 0.001, further validating the significance and reliability of the predictions. The 95% prediction intervals provide confidence ranges with nearly all data points falling within these intervals, with Cost predictions demonstrating particularly strong linear correlation, further confirming the reliability of the meta-model’s predictions. These analytical outcomes highlight the high precision of the meta-model when forecasting chemical benchmarks, which is crucial for predictive tasks in practical applications.

### 3.2 Sensitivity Analyses

In this section, the objective is to explore and quantify the impacts of various input variables on reaction time (RT), conversion rate (CR), and production cost (Cost) in ibuprofen synthesis. The CatBoost regression model combined with SHAP value analysis^41^ not only evaluates the model’s predictive capability but also reveals the relative importance of each variable and its specific contribution to prediction results. SHAP analysis demonstrates that catalyst concentration (*x*_5_) has the most significant influence on all three metrics, exhibiting complex nonlinear relationships—beyond a threshold concentration, the catalytic effect diminishes, reflecting a dynamic shift in the rate-limiting step within the catalytic cycle. Additionally, by plotting interaction effect diagrams, the study uncovers deeper mechanistic connections between input variables, such as the dual functionality of hydrogen ions in both the dehydration process and catalyst activation and how high catalyst concentrations can promote side reactions leading to decreased conversion rates. These analyses provide profound insights into reaction kinetics, revealing nonlinear mechanisms and multivariable optimization opportunities that transcend single-variable sensitivity analysis, offering deeper guidance for catalyst design and reaction condition optimization.

Figure 7 shows the feature importance analysis of different input variables on the reaction time (RT), conversion rate (CR), and production cost (Cost). For RT (Figure 7a), the most important feature is *x*_5_ (*c*_0_ (L_2_PdCl_2_)), followed by *x*_14_ (*k*_8_^r^), indicating that both catalyst precursor concentration and reverse reaction rate constant have dominant impacts on reaction time. The SHAP distribution for *x*_5_ exhibits clear nonlinear characteristics, suggesting that beyond a threshold concentration, the catalytic effect diminishes, reflecting a dynamic shift in the rate-limiting step within the catalytic cycle. *x*_3_ (*c*_0_ (H^+^)) and *x*_4_ (*c*_0_ (H_2_O)) also show significant influence, with the wide SHAP distribution for H^+^ revealing its dual functionality in both the dehydration process and catalyst activation mechanisms. For CR (Figure 7), *x*_5_ remains the most important feature, but its SHAP distribution reveals a complex negative correlation with conversion rate—exceeding the optimal concentration leads to decreased conversion, challenging conventional understanding. The negative SHAP values of *x*_13_ (*k*_8_^f^) indicate that side reactions (esterification) inhibit the product yield, with high catalyst concentrations potentially promoting these side reactions. For Cost (Figure 7), *x*_5_ and *x*_4_ are the most influential, with SHAP values spanning both positive and negative regions, indicating a complex cost-benefit relationship. Moderate catalyst levels improve economic efficiency by reducing reaction time, while excessive concentrations dramatically increase costs due to expensive palladium compounds. The impact of *x*_4_ (*c*_0_ (H_2_O)) likely relates to its role in the final hydrolysis step, forming ibuprofen, affecting both the reaction efficiency and subsequent purification costs. Overall, *x*_5_ is the most significant variable affecting RT, CR, and Cost, highlighting the importance of optimizing catalyst concentration to balance reaction efficiency with economic viability, while other features such as reactant concentration and rate constants also have significant impacts under specific conditions.

**Figure 7:**
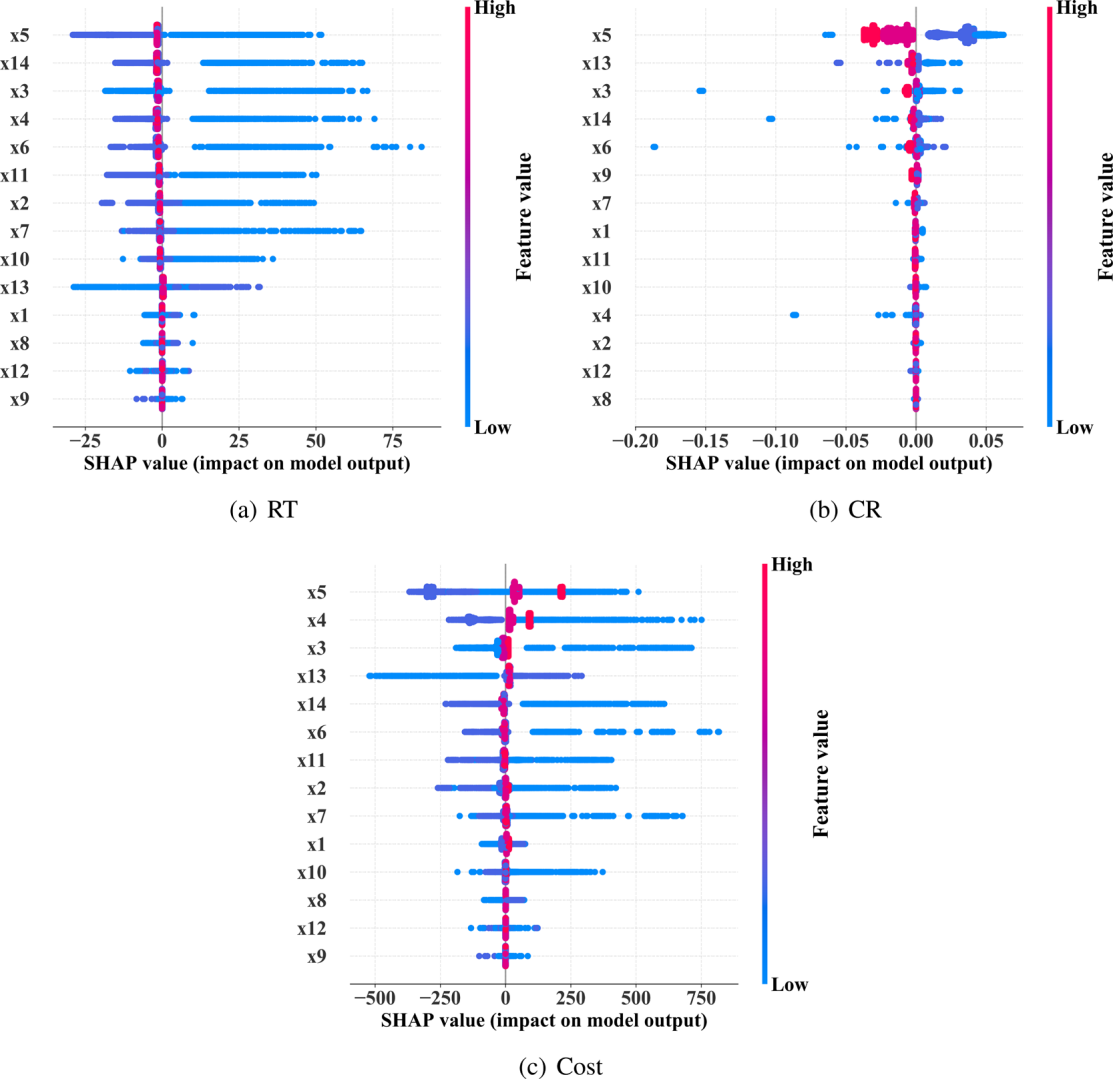
Feature importance analysis of individual features on RT, CR and Cost.

Figure 8 illustrates the dual-variable interaction effects on reaction time (RT), conversion rate (CR), and production cost (Cost), revealing critical mechanistic insights beyond traditional sensitivity analysis. For RT, Figure 8 demonstrates the interactions between the catalyst precursor concentration (*x*_5_) and hydrogen ion concentration (*x*_3_) or water concentration (*x*_4_), respectively. Both figures exhibit a pronounced threshold effect, where RT decreases dramatically when *x*_5_ exceeds a critical value and then stabilizes. This indicates that at low catalyst concentrations, the influences of *x*_3_ and *x*_4_ are significant, whereas at high catalyst concentrations, RT is predominantly controlled by catalyst concentration, with markedly diminished effects from other variables. From a mechanistic perspective, this phenomenon reveals a dynamic shift in the rate-limiting step: at low catalyst concentrations, catalyst activation (pd1 → pd2) is rate-limiting, while at high catalyst concentrations, the rate-limiting step shifts to carbonylation or hydrolysis steps. Particularly, in Figure 8, the contour distribution in the low *x*_5_ region indicates that water concentration significantly impacts the hydrolysis step (pd4 → ibu), a nonlinear effect difficult to capture with traditional kinetic analysis.

**Figure 8:**
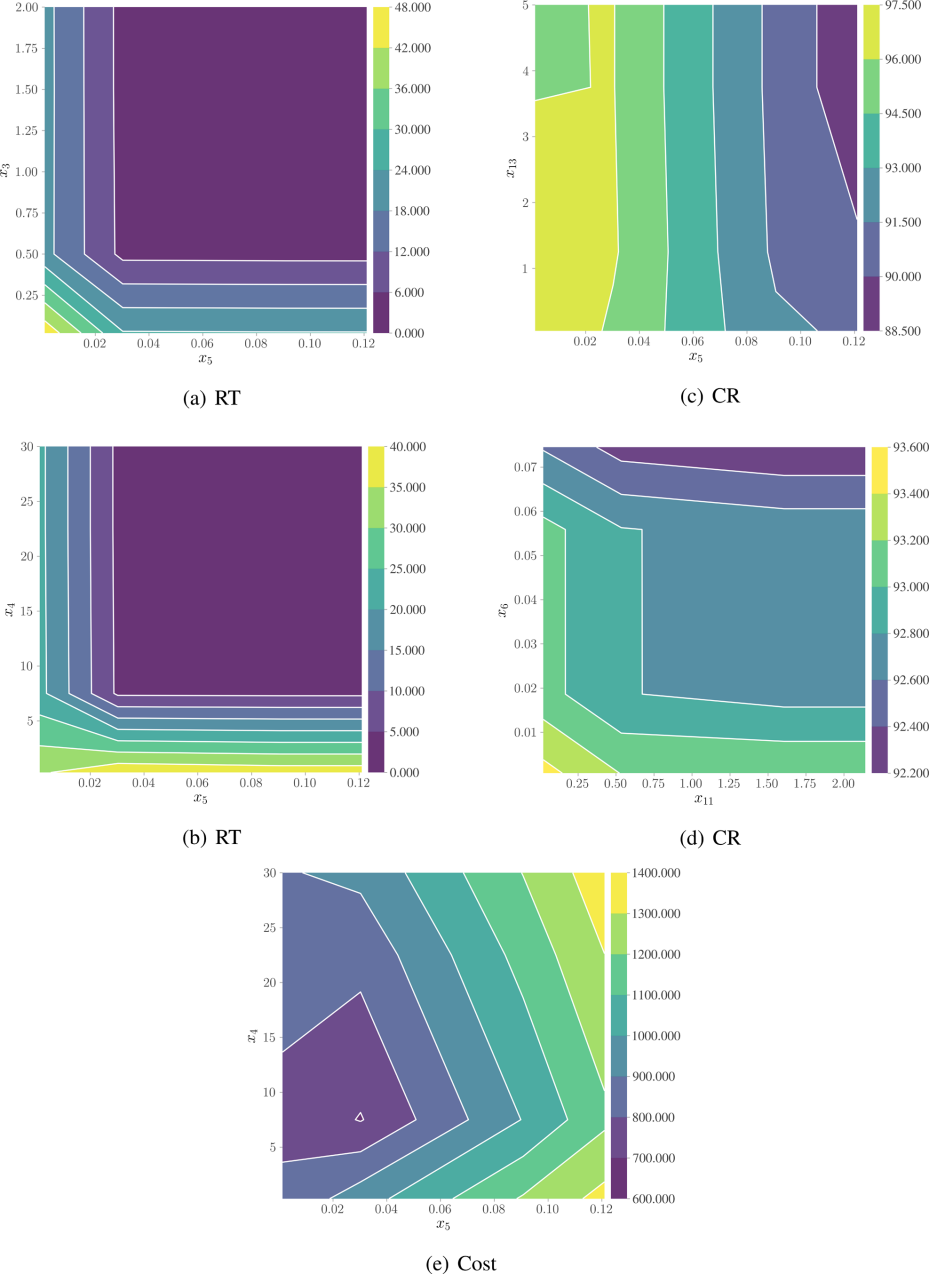
Mechanism-driven interaction effects of key variables on reaction performance metrics.

For CR, Figure 8 shows a complex interaction between the catalyst concentration (*x*_5_) and the side-reaction rate constant (*x*_13_). Surprisingly, higher catalyst concentrations correlate with lower conversion rates, as evidenced by the color bands progressively darkening from left to right (decreasing conversion), challenging the conventional notion that increasing catalyst concentration improves efficiency. This finding can be interpreted as high catalyst concentrations accelerating not only the main reaction but also competitive side reactions, particularly when the *x*_13_ (side-reaction rate constant) is elevated. Figure 8 reveals synergistic effects between the carbonylation step constant (*x*_11_) and the dehydration step constant (*x*_6_)—optimal conversion is achieved when both parameters are at moderate values. This discovery transcends optimization of single reaction steps, suggesting that catalyst design should consider the balance across multiple reaction stages simultaneously. For cost, Figure 8 demonstrates how the interaction between the catalyst concentration (*x*_5_) and water concentration (*x*_4_) influences the total production cost. The figure clearly identifies a minimum cost region at low catalyst concentrations and low-to-moderate water concentrations. The concave contour distribution indicates an optimal cost-efficiency combination rather than a simple linear relationship. This interaction pattern can be explained by the balance between catalyst cost and reaction efficiency, and the mechanism whereby water concentration affects hydrolysis step efficiency, subsequently impacting product purification costs.

### 3.3 Multiobjective Optimization

In this section, the NSGA-II algorithm is employed to perform multiobjective optimization of the ibuprofen synthesis process, simultaneously considering reaction time (RT), conversion rate (CR), and production cost (Cost) as three competing objectives. Based on the SHAP importance analysis results, key influencing factors are identified, and optimization parameter ranges are set according to Table 1. To ensure the optimization results have practical industrial application value, reasonable constraint conditions are set: reaction time between 0.5 and 72 h, conversion rate not less than 85% and not exceeding 98%, and production cost not exceeding 800 units. By generating 200 initial populations and optimizing through 500 generations of iterations, the Pareto optimal solution set is obtained, as shown in Figure 9, where each point represents a parameter combination that cannot be surpassed by other solutions simultaneously in all three objectives. Among all Pareto optimal solutions, a balanced performance solution is identified with reaction time RT = 4.28 h, conversion rate CR = 95.59%, and production cost Cost = 413.49, corresponding to optimal parameter values of hydrogen ion concentration (*x*_3_) = 1.59249414 mol/m^3^, catalyst concentration (*x*_5_) = 0.00449962 mol/m^3^, and dehydration reaction rate constant (*x*_6_) = 0.06696864 m^3^/(s mol).

**Figure 9:**
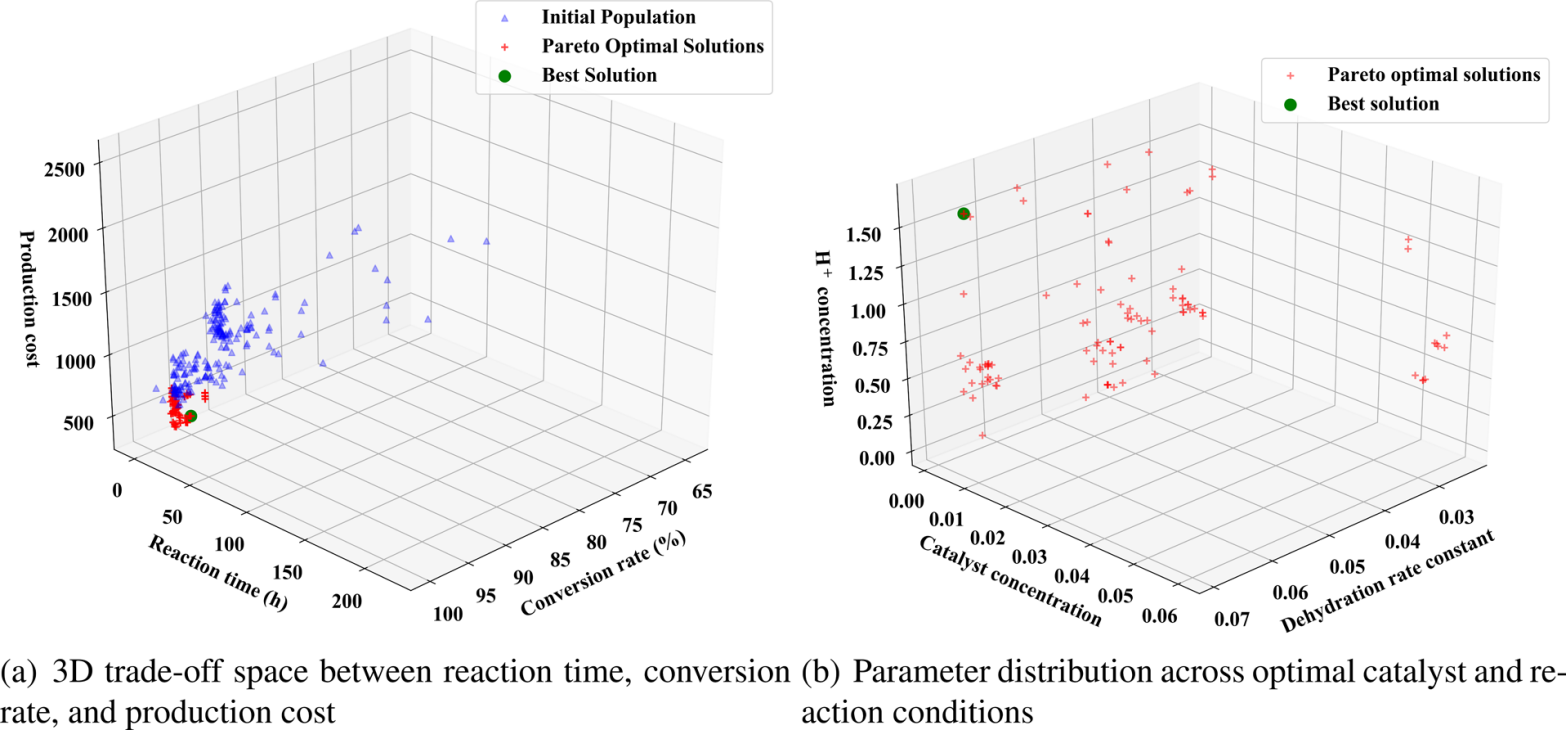
Pareto-optimal solutions for ibuprofen synthesis with multidimensional parameter space visualization.

Figure 9 shows the distribution of three key parameters corresponding to the Pareto optimal solutions, revealing the complex interaction relationships among catalyst concentration, hydrogen ion concentration, and dehydration reaction rate constant. Further analysis, as shown in Figure 10, indicates that catalyst concentration has a significant impact on conversion rate and cost: when the concentration is in the range of 0.002–0.01 mol/m^3^, high conversion rates of 95.5–98% can be achieved while maintaining relatively low costs; when the concentration increases to above 0.02 mol/m^3^, although reaction time can be shortened, the cost increases significantly with limited improvement in conversion rate. There exists a balance point between promoting the initial dehydration step and catalyst activation for hydrogen ion concentration, with the optimal balance strategy having a hydrogen ion concentration (1.59 mol/m^3^) that effectively promotes the dehydration reaction without affecting the catalyst stability. These complex nonlinear relationships explain why similar conversion rates can be achieved through different parameter combinations, reflecting the complexity of the ibuprofen synthesis reaction mechanism and the necessity of multiobjective optimization.

**Figure 10:**
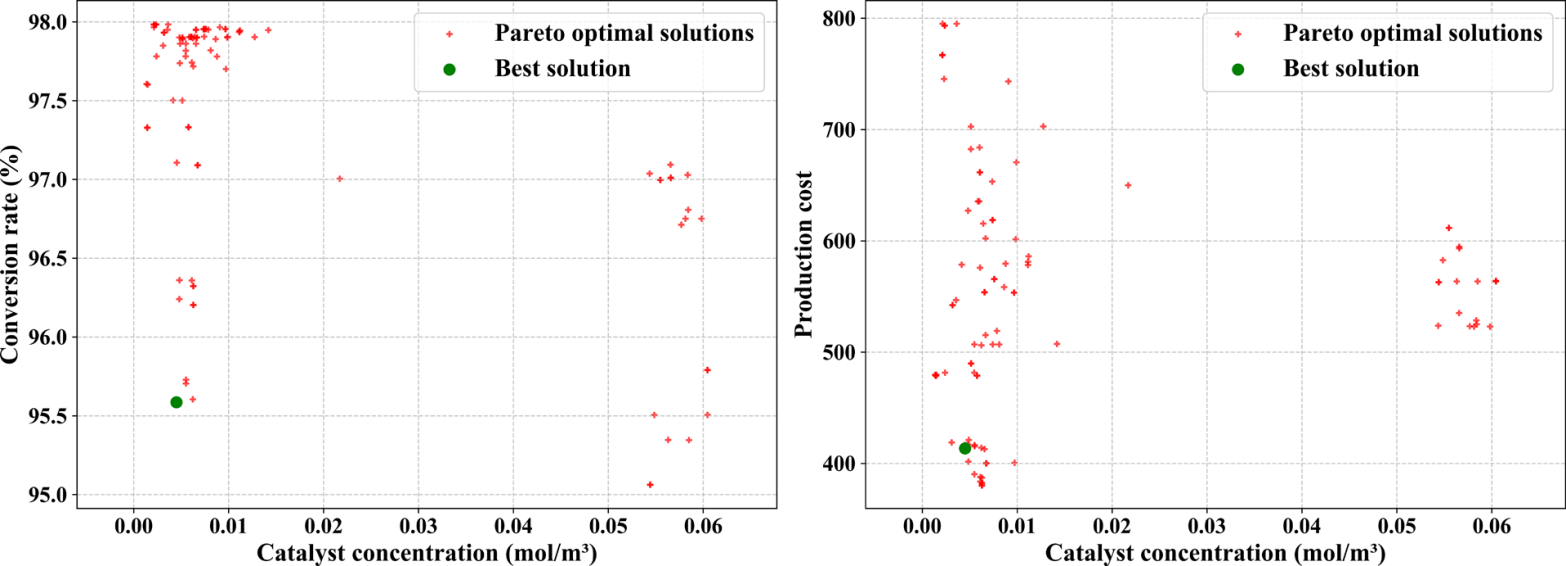
Impact of catalyst concentration on the conversion rate, reaction time, and production cost in ibuprofen synthesis.

Based on the multiobjective optimization results, four industrial strategies with clear application scenarios are identified. The radar chart in Figure 11 intuitively displays the trade-off characteristics of these strategies across the three objectives. The balanced performance strategy (RT = 4.28 h, CR = 95.59%, Cost = 413.49) achieves the best balance among the three objectives, suitable for regular stable production; the maximum output strategy (RT = 0.5 h, CR = 97.00%, Cost = 649.90) significantly shortens reaction time by increasing catalyst concentration (0.021694 mol/m^3^), suitable for peak demand periods; the maximum yield strategy (RT = 34.77 h, CR = 97.98%, Cost = 795.14) uses lower catalyst and hydrogen ion concentrations with longer reaction times, suitable for products requiring high purity; the minimum cost strategy (RT = 5.13 h, CR = 96.20%, Cost = 380.21) optimizes catalyst usage and reaction conditions to minimize production costs, suitable for large-scale or cost-sensitive markets. Similarity analysis shows that the balanced strategy and minimum cost strategy have high similarity (0.79), facilitating flexible switching in actual production. The above results demonstrate the practical application value of multiobjective optimization methods in complex chemical synthesis processes. While our optimization results provide computational insights for ibuprofen synthesis, direct comparison with commercial practices is limited by the proprietary nature of the industrial process data.

**Figure 11:**
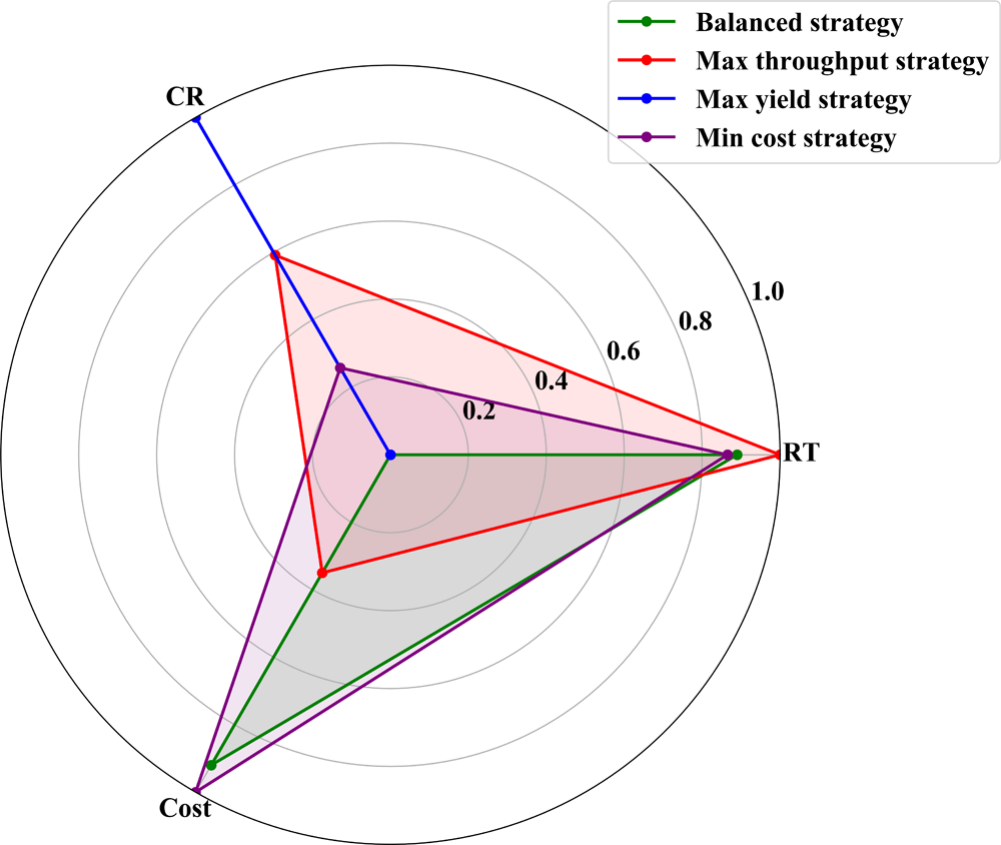
Radar chart comparing four industrial optimization strategies across reaction time, conversion rate, and cost objectives.

### 3.4 Uncertainty Analysis and Optimization Strategy Robustness

This section evaluates the sensitivity of ibuprofen synthesis to parameter fluctuations through Monte Carlo simulation, employing normal distribution *N*(0, σ × |*x*|) to generate perturbations. The σ values of 0.1, 0.2, 0.3, and 0.5 represent a systematic sensitivity analysis approach spanning mild to extreme parameter variation scenarios, providing methodological benchmarks for robustness assessment rather than site-specific industrial calibration. This approach enables standardized evaluation of the model stability across different perturbation intensities, where practitioners can adapt the method to their specific manufacturing contexts by substituting σ values derived from actual measurement uncertainties and control system capabilities. Each test sample undergoes 500 independent simulations, with perturbations simultaneously applied to all 14 input variables, including initial concentration parameters and reaction rate constants. Prediction uncertainty is quantified through 95% confidence intervals, while parameter interaction effects are visualized using radial basis function interpolation contour maps. The robustness assessment of optimization strategies is based on 30 independent simulations at σ = 0.2, employing differential perturbation weights for key parameters and a comprehensive scoring system considering both variation rates and coefficients of variation for RT, CR, and Cost metrics.

Comparative analysis in Figure 12 demonstrates that as perturbation levels increase, the prediction uncertainty of reaction time (RT) significantly exceeds that of conversion rate (CR) and production cost (Cost). Figure 13 further quantifies this relationship: RT uncertainty exhibits a distinctive nonlinear response, rising sharply from approximately 20% at σ = 0.1 to a peak of approximately 1700% at σ = 0.3, then declining to approximately 760% at σ = 0.5, while CR and Cost demonstrate nearly linear growth trends with notably different growth rates. This “bell-shaped” response curve for RT reveals that the system enters a critical state under moderate perturbations, a finding with important implications for industrial production, indicating that system stability is most vulnerable under parameter fluctuations at σ ≈ 0.3, requiring focused monitoring. The contour map in Figure 14 precisely characterizes the complex interaction effects between catalyst and hydrogen ion concentrations, with high-sensitivity regions primarily distributed in combinations of low catalyst (0–0.04 mol/m^3^) with high hydrogen ion (>1.4 mol/m^3^) concentrations, as well as in regions of higher catalyst concentration (0.11–0.13 mol/m^3^). This complex sensitivity distribution pattern provides clear parameter space guidance for optimizing reaction conditions, indicating that identified high-sensitivity regions should be avoided to ensure process stability.

**Figure 12:**
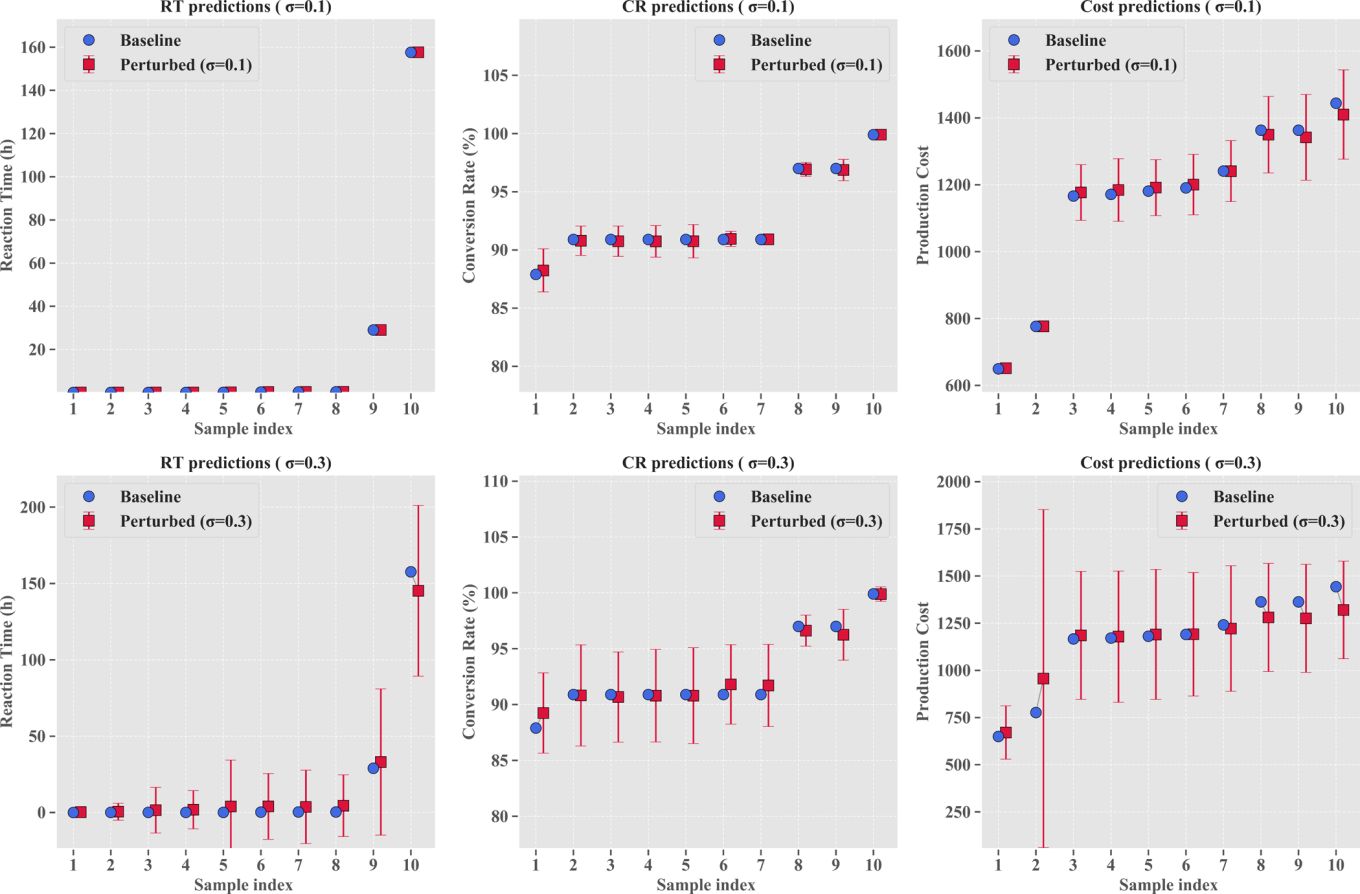
Comparison of baseline and perturbed predictions at different perturbation levels (σ = 0.1 and σ = 0.3) for the reaction time, conversion rate, and production cost.

**Figure 13:**
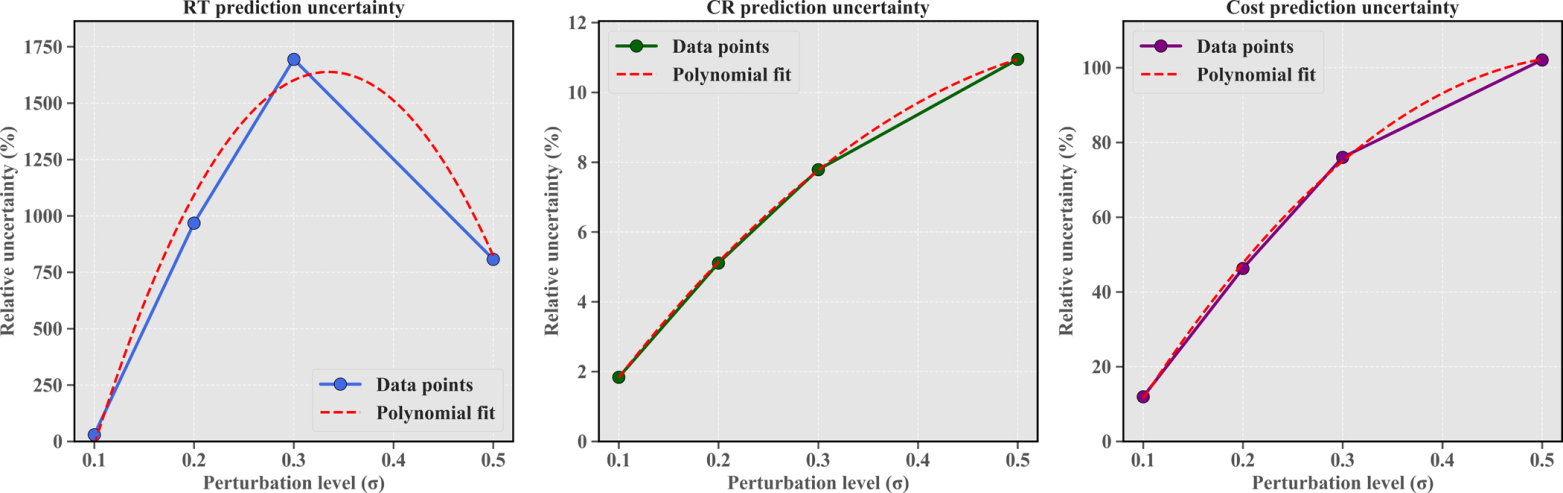
Quantitative relationship between the perturbation magnitude (σ) and relative prediction uncertainty (%) for the reaction time, conversion rate, and production cost metrics.

**Figure 14:**
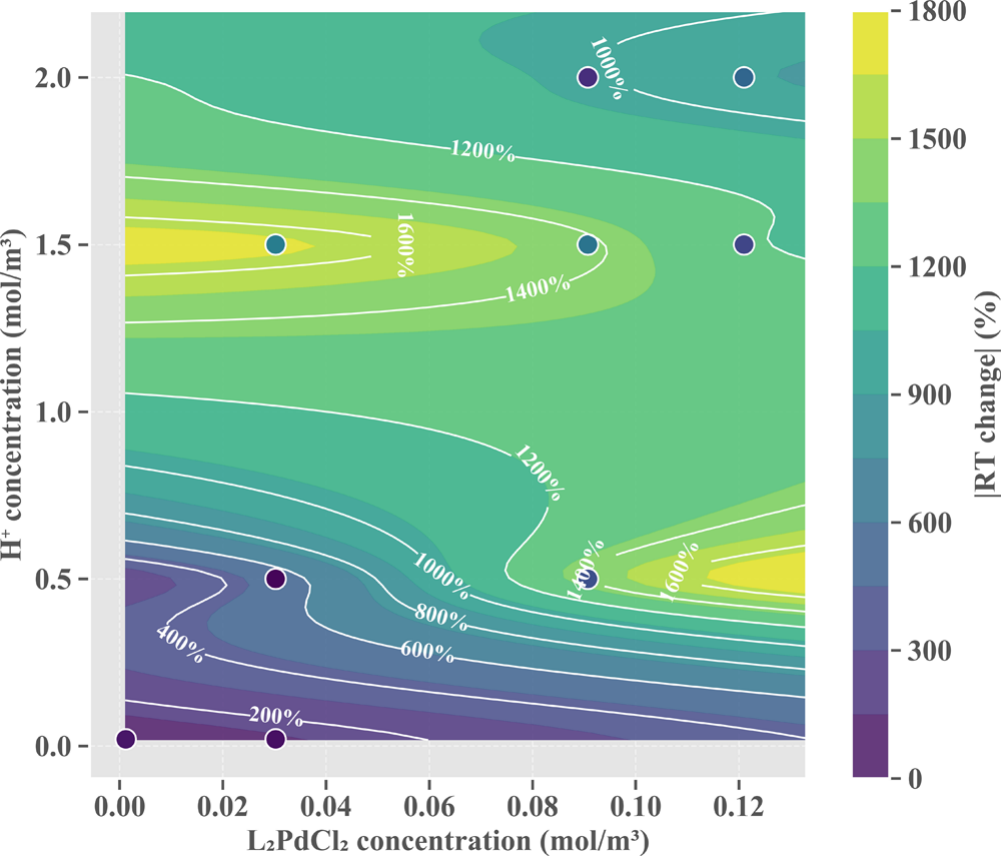
Parameter interaction effects between the L_2_PdCl_2_ catalyst concentration and H^+^ concentration on RT sensitivity at σ = 0.3.

Based on comprehensive robustness assessment results, the maximum yield strategy demonstrates optimal robustness (combined score 10.07), with relative variation rates of only 10.08 and 0.41% for RT and CR, respectively, significantly outperforming other strategies; in contrast, the maximum throughput strategy exhibits the lowest stability under parameter fluctuations, with an RT variation rate reaching 1045.76%, limiting its practical application value. According to the optimal strategy parameters obtained through multiobjective optimization, the recommendation for industrial production is to adopt the parameter combination of the maximum yield strategy, which is positioned in the low-sensitivity region of the parameter space, significantly enhancing production process robustness. Sensitivity testing further indicates that even under conditions where reaction kinetics parameters have systematic deviations of ±20%, the relative robustness ranking of optimization strategies remains consistent, verifying that the proposed method effectively addresses model uncertainties in actual production environments, providing a reliable scientific basis for robust design and optimization of ibuprofen synthesis processes.

## 4 Conclusions and Future Works

This study demonstrates the application of integrated machine learning and optimization techniques for ibuprofen synthesis process optimization by combining physical models with data-driven approaches, achieving reasonable prediction accuracy for complex chemical reactions. A detailed simulation model implemented in COMSOL Multiphysics software establishes a database to explore the catalytic reaction dynamics of ibuprofen synthesis, meticulously tracking each reaction step from the dehydration of 1-(4-isobutylphenyl)­ethanol to the formation of ibuprofen. The results indicate that the CatBoost model predictions of reaction time (RT), conversion rate (CR), and production cost (Cost) closely align with actual chemical outcomes, with 95% confidence intervals demonstrating the model’s high reliability. Comparative analysis with traditional optimization algorithms (RS, Bayesian, PSO, and SA) reveals that the SAO-CatBoost framework exhibits a superior predictive accuracy. Sensitivity analysis shows that the concentration of the catalyst precursor (L_2_PdCl_2_) is the most critical factor affecting RT, CR, and Cost, with other significant variables including reaction rate constants and hydrogen ion concentration. SHAP value analysis reveals nonlinear impact characteristics among key input variables: the catalytic effect diminishes beyond threshold concentrations, reflecting dynamic changes in rate-limiting steps within the catalytic cycle; hydrogen ions demonstrate dual functionality in dehydration processes and catalyst activation mechanisms; and high catalyst concentrations may promote side reactions leading to decreased conversion rates. These quantified relationships validate known catalytic principles while providing systematic parameter guidance for optimization. However, since the framework is validated using simulation data, experimental verification is necessary to confirm the practical applicability of the optimization strategies.

Through multiobjective optimization, the study identifies several ibuprofen synthesis strategies applicable to different industrial scenarios: the balanced performance strategy (RT = 4.28 h, CR = 95.59%, Cost = 413.49) achieves optimal balance among the three objectives, suitable for regular stable production; the maximum output strategy (RT = 0.5 h, CR = 97.00%, Cost = 649.90) significantly shortens reaction time by increasing catalyst concentration (0.021694 mol/m^3^), suitable for peak demand periods; the maximum yield strategy (RT = 34.77 h, CR = 97.98%, Cost = 795.14) employs lower catalyst and hydrogen ion concentrations with longer reaction times, suitable for products requiring high purity; and the minimum cost strategy (RT = 5.13 h, CR = 96.20%, Cost = 380.21) optimizes catalyst usage and reaction conditions to minimize production costs, suitable for large-scale or cost-sensitive markets. Multiobjective optimization results indicate that catalyst concentrations in the range of 0.002–0.01 mol/m^3^ can achieve high conversion rates of 95.5–98% while maintaining relatively low costs, whereas when concentrations increase to above 0.02 mol/m^3^, although reaction times are shortened, costs increase significantly with limited improvement in conversion rates. Systematic uncertainty analysis shows that as perturbation levels increase, prediction ranges expand significantly, particularly for RT predictions, indicating that input variable fluctuations have a substantial impact on prediction uncertainty. Robustness assessment results demonstrate that the maximum yield strategy exhibits optimal robustness under parameter fluctuations (σ = 0.2) with a combined score of 10.07.

This work is based on COMSOL simulation data for model development and validation. While the simulation model shows good agreement with limited experimental data, the optimization strategies require laboratory-scale experimental validation before practical implementation to ensure their effectiveness in real chemical systems. This is because simulation-based approaches inherently cannot capture critical industrial factors, such as catalyst deactivation, equipment-specific mass and heat transfer effects, process variability, raw material quality fluctuations, and scale-up challenges. Future work will focus on: experimental validation of the four identified optimization strategies using batch reactor studies to compare predicted versus actual reaction time, conversion rate, and production cost, with targeted experimental testing of optimal catalyst concentration ranges (0.002–0.01 mol/m^3^) and high-sensitivity parameter combinations identified through uncertainty analysis. This research contributes to demonstrating how systematic integration of machine learning and optimization techniques can be applied to chemical process optimization, providing a practical methodology for chemical manufacturing applications. Additionally, future studies will explore the application of this integrated approach in other chemical reactions and industrial processes to further validate its applicability.

## Supplementary Material



## Data Availability

The data underlying this study are not publicly available due to the large size and complexity of the data sets requiring specific processing and interpretation guidance. The data are available from the corresponding author upon reasonable request.

## Notes

Kinetic modeling details for ibuprofen synthesis reactions (Text S1); snow ablation optimizer (SAO) algorithm description and pseudocode (Text S2); categorical boosting (CatBoost) model methodology (Text S3); meta-model evaluation metrics and formulations (Text S4); NSGA-II optimization process details (Text S5); catalytic mechanism and process diagram (Figure S1); SAO algorithm pseudocode (Algorithm S1); time complexity analysis of SAO (Algorithm S2); and unbiased gradient estimation algorithm (Algorithm S3) (PDF)
