# Supplementary material for: Kinetic Modeling and Multiobjective Optimization of Ibuprofen Synthesis Using Machine Learning
Source: ACS Omega. 2025 Aug 8;10(32):35512–27. doi: 10.1021/acsomega.4c11725 (PMC12368723; doi:10.1021/acsomega.4c11725)
Supplement: Supplementary file 1 [file ao4c11725_si_001.pdf]

Supporting Information

Kinetic modeling and multi-objective optimization of ibuprofen synthesis using machine learning

Lang Xiang[a], Pengfei Qu[b,*]

[a]*School of Pharmacy, Nanjing University of Chinese Medicine, Nanjing, China*
[b]*School of Management Science and Engineering, Shandong Technology and Business University, Yantai 264005, China*

Table of Contents

Text S1. Kinetic modeling of ibuprofen synthesis S1

Text S2. Snow ablation optimizer (SAO) S4

Text S3. Categorical boosting (CatBoost) model S7

Text S4. Meta-model evaluation S9

Text S5. NSGA-II optimization process S11

Figure S1. Catalytic mechanism and process of ibuprofen synthesis S2

Algorithm S1. Snow Ablation Optimizer (SAO) S5

Algorithm S2. Time complexity of SAO S7

Algorithm S3. Unbiased gradient estimation S9

References S13

Text S1. Kinetic modeling of ibuprofen synthesis

Refinement in the modeling of ibuprofen synthesis is dependent on an in-depth comprehension of the reaction steps, kinetic behaviors, and the entirety of the catalytic cycle, with particular

*Corresponding author
Email address: qupengfeiyt@163.com (Pengfei Qu)

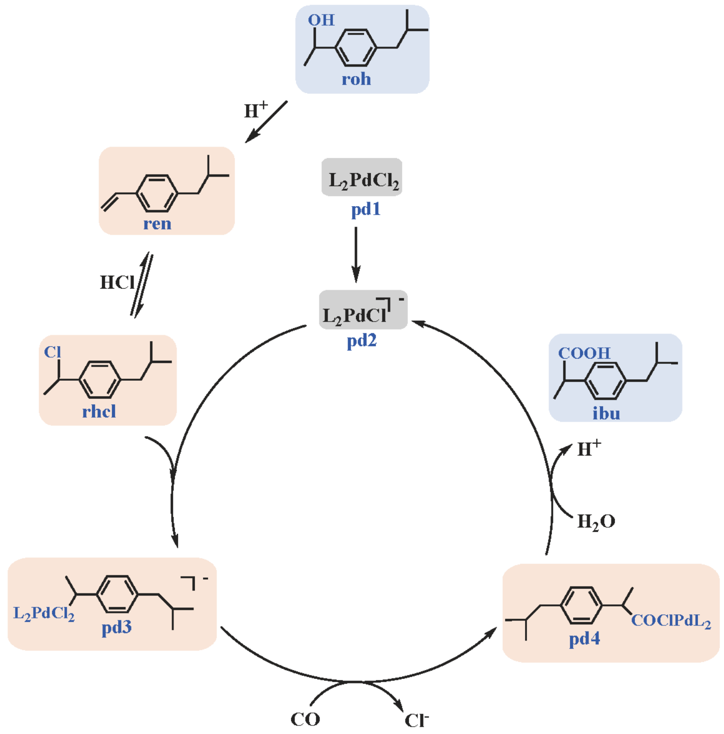

Figure S1: Catalytic mechanism and process of ibuprofen synthesis.

emphasis on the interactions and transformations among reactants. Mastery of these elements is indispensable for the construction of a model that accurately reflects the chemical process. Enhanced modeling precision is instrumental in improving predictions of reaction dynamics, optimizing manufacturing processes, and augmenting both the yield and quality of ibuprofen. Figure S1 provides a detailed illustration of the catalytic cycle, offering crucial visual support for modeling endeavors.

As depicted in Figure S1, the synthesis of ibuprofen begins with the dehydration of the initial substance, 1-(4-isobutylphenyl)ethanol, to produce 4-isobutylstyrene. This intermediate then reacts with hydrochloric acid to yield the active intermediate, 1-(4-isobutylphenyl)ethyl chloride. Following this, the palladium catalyst, initially in the form of L_2PdCl_2 (where L denotes triphenylphosphine), transforms into its anionic version, L_2PdCl, becoming catalytically active. The now-active catalyst promotes the carbonylation and subsequent hydrolysis of 1-(4-isobutylphenyl)ethyl chloride, culminating in the production of ibuprofen.

The chemical equations for the catalytic cycle of ibuprofen synthesis are as follows[1]:

$$\mathrm{roh} + \mathrm{H}^+ \rightarrow \mathrm{ren} + \mathrm{H_2O} + \mathrm{H}^+ \tag{S1}$$

$$\mathrm{ren} + \mathrm{H}^+ + \mathrm{Cl}^- \rightarrow \mathrm{rhcl} \tag{S2}$$

$$\mathrm{rhcl} + \mathrm{B} \rightarrow \mathrm{ren} + \mathrm{H}^+ + \mathrm{Cl}^- + \mathrm{B} \tag{S3}$$

$$\mathrm{pd1} + \mathrm{CO} + \mathrm{H_2O} \rightarrow \mathrm{pd2} + \mathrm{Cl}^- + 2\mathrm{H}^+ + \mathrm{CO_2} \tag{S4}$$

$$\mathrm{pd2} + \mathrm{rhcl} \rightarrow \mathrm{pd3} \tag{S5}$$

$$\mathrm{pd3} + \mathrm{CO} \rightarrow \mathrm{pd4} + \mathrm{Cl}^- \tag{S6}$$

$$\mathrm{pd4} + \mathrm{H_2O} \rightarrow \mathrm{pd2} + \mathrm{H}^+ + \mathrm{ibu} \tag{S7}$$

The first reaction ($\mathrm{roh} + \mathrm{H}^+$) involves dehydrating the reactant alcohol to yield the corresponding alkene (ren). The second reaction ($\mathrm{ren} + \mathrm{H}^+ + \mathrm{Cl}^-$) involves the alkene undergoing hydrohalogenation, forming the active substrate (rhcl). In the third reaction (rhcl + B), this active substrate undergoes dehydrohalogenation with the help of a base, B. The fourth reaction ($\mathrm{pd1} + \mathrm{CO} + \mathrm{H_2O}$) transforms the precatalyst (pd1) into the active anionic catalyst (pd2). During the fifth reaction (pd2 + rhcl), the active substrate is subjected to oxidative addition to the L2PdCl catalyst (pd3). The sixth reaction (pd3 + CO) encompasses the carbonylation process (pd4), and the seventh and final reaction ($\mathrm{pd4} + \mathrm{H_2O}$) describes the hydrolysis of the metalorganic compound, which results in the production of ibuprofen (ibu) and the regeneration of the catalyst (pd2).

The reaction rates for reactions 1 to 7 can be expressed as follows:

$$v_1 = k_1 c_{\mathrm{roh}} c_{\mathrm{H}} \tag{S8}$$

$$v_2 = k_2 c_{\mathrm{ren}} c_{\mathrm{H}} c_{\mathrm{Cl}} \tag{S9}$$

$$v_3 = k_3 c_{\text{rhcl}} c_{\text{B}} \tag{S10}$$

$$v_4 = k_4 c_{\text{pd1}} c_{\text{CO}} c_{\text{H}_2\text{O}} \tag{S11}$$

$$v_5 = k_5 c_{\text{pd2}} c_{\text{rhcl}} \tag{S12}$$

$$v_6 = k_6 c_{\text{pd3}} c_{\text{CO}} \tag{S13}$$

$$v_7 = k_7 c_{\text{pd4}} c_{\text{H}_2\text{O}} \tag{S14}$$

where k represents the reaction rate constant for each respective reaction.

Text S2. Snow ablation optimizer (SAO)

The Snow Ablation Optimizer (SAO) [2] is an innovative metaheuristic algorithm inspired by the natural process of snow ablation, which includes both sublimation (direct transition from snow to vapor) and melting (transition from snow to water). This algorithm achieves a balance between exploration and exploitation in the search space, preventing premature convergence to local optima. The SAO algorithm is particularly suitable for solving complex numerical optimization and engineering design problems, such as those involving nonlinearity, nonsmoothness, and dynamic characteristics. Extensive benchmark tests and real-world applications have demonstrated that SAO exhibits significant competitiveness and efficiency compared to other advanced metaheuristic algorithms. The pseudocode is displayed in Algorithm S1.

Algorithm S 1: Snow Ablation Optimizer (SAO)

Input: Population Z_i ($i = 1, 2, \ldots, N$), Maximum iterations t_{max}

Output: Best solution $G(t)$

```
Initialize population: Z_i;
Evaluate initial fitness of the population;
Record the best individual as G(t);
while t < t_max do
    Calculate snowmelt rate M using the degree-day factor;
    Randomly split the population P into two subpopulations P_a and P_b;
    for each individual i do
        Update the position of individual i based on the subpopulation's strategy;
    end
    Evaluate the fitness of the updated population;
    Update the best solution G(t);
    Increment iteration counter t;
end
return Best solution G(t);
```

In SAO, the algorithm first initializes the population and parameters, then evaluates the initial fitness of the population. During each iteration, the snowmelt rate is calculated, and the population is randomly divided into two subpopulations for exploration and exploitation. After updating the positions of individuals, the fitness is re-evaluated, and the best solution is updated. This process continues until the maximum number of iterations is reached, and the best solution found is returned.

In the initialization phase, the algorithm randomly generates a population of size N, where each individual's position is represented as an $N \times \text{Dim}$ matrix, with Dim being the dimension of

the problem. The initialization formula is as follows:

$$Z = L + \theta \times (U - L) \tag{S15}$$

where L and U represent the lower and upper bounds of the solution space, respectively, and θ is a randomly generated number in the interval $[0, 1]$.

In the exploration phase, the sublimation process of snow is simulated, where individuals expand to different regions of the solution space through irregular movements. Brownian motion is used to model this high-dispersal characteristic. The position update formula is as follows:

$$Z_i(t+1) = \text{Elite}(t) + BM_i(t) \otimes (\theta_1 \times (G(t) - Z_i(t)) + (1 - \theta_1) \times (\overline{Z}(t) - Z_i(t))) \tag{S16}$$

where $Z_i(t)$ represents the position of the i-th individual at the t-th iteration, $BM_i(t)$ is a random vector based on Gaussian distribution, θ_1 is a random number in the interval $[0, 1]$, $G(t)$ represents the current best solution, $\overline{Z}(t)$ is the centroid position of the population, and Elite(t) is a randomly selected individual from the elite members of the population.

In the exploitation phase, the melting process of snow is simulated, where individuals perform local searches around the current best solution. The snowmelt rate M is calculated as follows:

$$M = \text{DDF} \times T \tag{S17}$$

where T is the average daily temperature, and DDF is the degree-day factor, updated according to:

$$\text{DDF} = 0.35 + 0.25 \times \frac{e^{\frac{t}{t_{\max}}} - 1}{e - 1} \tag{S18}$$

The position update formula in this phase is:

$$Z_i(t+1) = M \times G(t) + BM_i(t) \otimes (\theta_2 \times (G(t) - Z_i(t)) + (1-\theta_2) \times (\overline{Z}(t) - Z_i(t))) \quad \text{(S19)}$$

where θ_2 is a random number in the interval $[-1, 1]$.

To balance exploitation and exploration, SAO employs a dual-population mechanism. The entire population is randomly divided into two subpopulations at the early stages, with one focusing on exploration and the other on exploitation. As iterations proceed, the size of the exploration subpopulation gradually increases, while the size of the exploitation subpopulation decreases.

Algorithm S 2: Time complexity of SAO

Input: Population size N, Dimension Dim, Iterations t

Output: Total time complexity

1 Initialization: $O(N \times \text{Dim})$;

2 Position updating: $O(N \times \text{Dim})$;

3 Fitness evaluation: $O(N)$;

4 Fitness sorting: $O(N \times \log N)$;

5 Total time complexity: $O(N \times \text{Dim} + N \times t \times (\log N + \text{Dim} + 1))$;

This algorithm's time complexity includes components such as initialization, position updating, fitness evaluation, and fitness sorting. The total time complexity is estimated as shown in Algorithm S2. The above analysis demonstrates that SAO is a well-structured and efficient new metaheuristic algorithm capable of effectively solving complex optimization problems.

Text S3. Categorical boosting (CatBoost) model

CatBoost is a high-performance open-source gradient boosting algorithm designed specifically for handling categorical features. It outperforms existing gradient boosting implementations, such as XGBoost and LightGBM, on various public datasets [3]. CatBoost is particularly suitable for datasets with heterogeneous features and complex dependencies, such as web search, recommen-

dation systems, and weather forecasting. By processing categorical features during training rather than at the preprocessing stage, CatBoost significantly reduces the risk of overfitting. Additionally, its novel leaf value calculation method further enhances model stability and predictive accuracy. CatBoost offers both GPU and CPU implementations, with the GPU version greatly accelerating training speed and the CPU version excelling in model scoring.

The core technique in CatBoost for handling categorical features involves randomly permuting the dataset and replacing the categorical feature value of the current sample with the average label value of preceding samples with the same categorical value. The specific equation is:

$$x_{\sigma_p,k} = \frac{\sum_{j=1}^{p-1}[x_{\sigma_j,k} = x_{\sigma_p,k}] \cdot Y_{\sigma_j} + a \cdot P}{\sum_{j=1}^{p-1}[x_{\sigma_j,k} = x_{\sigma_p,k}] + a} \tag{S20}$$

where σ represents a permutation of the dataset indices, P is a prior value, and a is the weight of the prior. This method effectively reduces overfitting by incorporating prior knowledge and using part of the data to compute statistics.

To address the gradient bias problem, CatBoost uses unbiased gradient estimation as shown in Algorithm S3. Traditional gradient boosting methods estimate gradients using the same data points at each step, leading to biased gradient estimates and overfitting. CatBoost mitigates this issue by training separate models to estimate the gradient for each sample, thus reducing the bias.

Algorithm S 3: Unbiased gradient estimation

```
Input: Dataset {X_k, Y_k} ordered by permutation σ, number of trees I
Initialize models M_i = 0 for i = 1 to n;
for iter = 1 to I do
    for i = 1 to n do
        for j = 1 to i − 1 do
            g_j = d/da Loss(Y_j, a)|_{a=M_i(X_j)};
        end
        M = LearnOneTree({X_j, g_j} for j = 1 to i − 1);
        M_i = M_i + M;
    end
end
return models M_1, ..., M_n
```

CatBoost utilizes oblivious trees (symmetric trees) to maintain balanced tree structures and reduce overfitting. These trees use the same splitting criterion across all levels, enhancing model stability. Additionally, CatBoost accelerates GPU training through feature discretization and histogram-based approaches.

CatBoost, with its innovative categorical feature handling, unbiased gradient estimation, and efficient implementation, excels in processing datasets with numerous categorical features. It outperforms existing gradient boosting libraries like XGBoost and LightGBM in both speed and predictive accuracy.

Text S4. Meta-model evaluation

In model evaluation, the Mean Squared Error (MSE), Root Mean Squared Error (RMSE), Mean Absolute Error (MAE), Coefficient of Determination (R^2), and Mean Absolute Percentage Error (MAPE) are five commonly used statistical metrics. Each metric has its unique character-

istics and application scope. These metrics, when used together, can comprehensively assess the performance of a model, revealing prediction errors and fitting accuracy from different perspectives. By combining these indicators, it is possible to analyze the overall error level of the model and identify its strengths and weaknesses in specific situations, thus providing a basis for model optimization.

MSE measures the average error of the model by calculating the mean of the squared differences between predicted and actual values. A lower MSE indicates higher predictive accuracy of the model. The calculation method is as follows [4]:

$$\mathrm{MSE} = \frac{1}{n}\sum_{i=1}^{n}(A_i - P_i)^2 \tag{S21}$$

where A_i is the actual value, P_i is the predicted value, and n is the number of observations.

RMSE is the square root of MSE, which retains the same unit as the original data, making it easier to interpret. The calculation method is as follows [5]:

$$\mathrm{RMSE} = \sqrt{\frac{1}{n}\sum_{i=1}^{n}(A_i - P_i)^2} \tag{S22}$$

where A_i is the actual value, P_i is the predicted value, and n is the number of observations.

MAE measures the average error by calculating the mean of the absolute differences between predicted and actual values. The calculation method is as follows [5]:

$$\mathrm{MAE} = \frac{1}{n}\sum_{i=1}^{n}|A_i - P_i| \tag{S23}$$

where A_i is the actual value, P_i is the predicted value, and n is the number of observations.

R^2 reflects the goodness of fit of the model to the actual data, with a range from 0 to 1. The closer the R^2 value is to 1, the better the model explains the variability of the data. The calculation

method is as follows [6]:

$$R^2 = 1 - \frac{\sum_{i=1}^{n}(A_i - P_i)^2}{\sum_{i=1}^{n}(A_i - \bar{A})^2} \tag{S24}$$

where A_i is the actual value, P_i is the predicted value, $\bar{A}$ is the mean of the actual values, and n is the number of observations.

MAPE measures the average prediction error as a percentage of the actual values. The calculation method is as follows [6]:

$$\text{MAPE} = \frac{1}{n}\sum_{i=1}^{n}\left|\frac{A_i - P_i}{A_i}\right| \times 100\% \tag{S25}$$

where A_i is the actual value, P_i is the predicted value, and n is the number of observations.

By using these metrics, the performance of the model can be comprehensively evaluated from multiple angles, helping to select the optimal model and improve prediction algorithms.

Text S5. NSGA-II optimization process

NSGA-II (Non-dominated Sorting Genetic Algorithm II) is an evolutionary algorithm designed for multi-objective optimization [7]. Compared to its predecessor NSGA, NSGA-II significantly improves in terms of computational complexity, elitism, and the need for a sharing parameter. Specifically, NSGA-II reduces the computational complexity from $O(mN^3)$ to $O(mN^2)$, introduces an elitist approach to ensure the retention of superior individuals, and eliminates the need for a sharing parameter. The algorithm achieves effective Pareto front identification while maintaining population diversity through fast non-dominated sorting and crowding distance calculation. NSGA-II is widely applied in complex engineering and scientific problems requiring simultaneous optimization of multiple objectives, such as design optimization, resource allocation, and scheduling.

Non-dominated sorting is used to classify the population into different levels of non-domination. For each individual p in the population, the number of individuals dominating p (n_p) and the set

of individuals dominated by p (S_p) are calculated:

$$n_p = \sum_{q \in P} I(q \prec p) \tag{S26}$$

$$S_p = \{q \in P \mid p \prec q\} \tag{S27}$$

where I is an indicator function that equals 1 if the condition is true and 0 otherwise; $p \prec q$ denotes that individual p dominates individual q. Initially, all individuals with $n_p = 0$ are placed in the first front F_1. Subsequent fronts are generated by updating n_q and processing each front F_i.

The crowding distance is used to estimate the density of solutions surrounding a particular individual, ensuring population diversity. The calculation method is as follows:

$$d_i = \sum_{m=1}^{M} \frac{f_{i+1}^m - f_{i-1}^m}{f_{\max}^m - f_{\min}^m} \tag{S28}$$

where f_i^m denotes the value of the i-th individual in the m-th objective, and $f_{\max}^m$ and $f_{\min}^m$ are the maximum and minimum values of the m-th objective, respectively. The crowding distance is obtained by sorting the population for each objective and calculating the distance between neighboring individuals.

The parent and offspring populations are merged, and the top N individuals are selected based on non-dominated sorting and crowding distance to form the next generation. First, the populations are combined $R_t = P_t \cup Q_t$. The combined population is then sorted using the fast non-dominated sorting algorithm, producing multiple fronts $F_1, F_2, \ldots$. Individuals from these fronts are added to the new population until the population size N is reached. If the last front exceeds N, individuals are selected based on crowding distance to fill the population.

References

[1] Kjonaas, R.A., Williams, P.E., Counce, D.A., Crawley, L.R.. Synthesis of ibuprofen in the introductory organic laboratory. Journal of Chemical Education 2011;88(6):825–828.

[2] Deng, L., Liu, S.. Snow ablation optimizer: A novel metaheuristic technique for numerical optimization and engineering design. Expert Systems with Applications 2023;225:120069.

[3] Dorogush, A.V., Ershov, V., Gulin, A.. Catboost: gradient boosting with categorical features support. arXiv preprint arXiv:181011363 2018;.

[4] Köksoy, O.. Multiresponse robust design: Mean square error (mse) criterion. Applied Mathematics and Computation 2006;175(2):1716–1729.

[5] Chai, T., Draxler, R.R., et al. Root mean square error (rmse) or mean absolute error (mae). Geoscientific model development discussions 2014;7(1):1525–1534.

[6] Chicco, D., Warrens, M.J., Jurman, G.. The coefficient of determination r-squared is more informative than smape, mae, mape, mse and rmse in regression analysis evaluation. Peerj computer science 2021;7:e623.

[7] Deb, K., Agrawal, S., Pratap, A., Meyarivan, T.. A fast elitist non-dominated sorting genetic algorithm for multi-objective optimization: Nsga-ii. In: Parallel Problem Solving from Nature PPSN VI: 6th International Conference Paris, France, September 18–20, 2000 Proceedings 6. Springer; 2000, p. 849–858.
